# Reducing sugars: a potential factor for onion plant salinity adaptation

**DOI:** 10.1186/s12870-025-07722-0

**Published:** 2025-11-26

**Authors:** Nesma Nabil Ibrahim Mohamed, Mahmoud M. Ghuniem, Gamal S. Khalifa, Mohamed A. A. Mahmoud, Hemmat A. Ibrahim

**Affiliations:** 1 Agricultural Biochemistry Department, Faculty of Agriculture, Ain Shams University, P.O. Box 68, HadayekShobra, 11241 Cairo Egypt; 2 Ministry of Agriculture and Land Reclamation, Agricultural Research Center (ARC), Central Laboratory of Residue Analysis of Pesticides and Heavy Metals in Food (QCAP), 7 Nadi Elsaid St, Dokki, 12311 Giza, Egypt; 3 Agricultural Botany Department, Faculty of Agriculture, Ain Shams University, P.O. Box 68, HadayekShobra, 11241 Cairo Egypt

**Keywords:** Salinity stress, Allium cepa, Reducing sugars, Oxidative stress, Osmoprotectants, Volatile sulfur compounds

## Abstract

Salinity stress critically impairs agricultural productivity by disrupting plant growth, nutrient homeostasis, and oxidative balance. Although sugars contribute to osmoprotection, their specific role in onion (*Allium cepa* L.) salinity adaptation remains underexplored. This study evaluated the effects of foliar applications of reducing sugars (glucose, lactose, melibiose, and xylose at 10 mM) on the physiological, biochemical, and elemental responses of pot-grown onions exposed to salinity stress (0, 75, and 125 mM NaCl). Growth parameters, photosynthetic pigments, osmoprotectants, oxidative stress markers, antioxidant enzymes, elemental composition, and volatile compounds were analyzed. At 125 mM NaCl, salinity reduced plant height by 29.45% and fresh weight by 63.33%. Sugar treatments markedly alleviated these effects, with lactose showing the greatest improvement in fresh weight (189.09% increase). Photosynthetic pigments were preserved, notably chlorophyll a by xylose (558.04 mg 100 g⁻¹ FW) and chlorophyll b by melibiose (272.65 mg 100 g⁻¹ FW). Osmoprotectants increased under sugar application, with melibiose enhancing proline (8.93 mg 100 g⁻¹ FW) and xylose elevating reducing sugars (229.16 mg 100 g⁻¹ FW). Lactose lowered oxidative stress (H₂O₂: 198.19 mg 100 g⁻¹; MDA: 0.00075 µmol g⁻¹ FW) while enhancing PAL (9314 U mg⁻¹ protein) and PPO (26 U mg⁻¹ protein) activities. Sugar treatments also influenced ion homeostasis, with melibiose and lactose improving Ca²⁺/Na⁺ and K⁺/Na⁺ ratios. Elemental intake assessment showed that most essential elements remained within safe limits, although Fe and Mn slightly exceeded dietary thresholds in a few treatments. These findings identify lactose and melibiose as promising foliar treatments for enhancing onion salinity tolerance through improved osmoprotection, antioxidant defense, and mineral regulation.

## Background

 Salinity stress is a critical challenge to agricultural productivity, affecting over 900 million hectares globally, with one-third of this land under cultivation [1, 2]. Salinity stress impairs plant growth by reducing water uptake, causing ion toxicity, and disrupting nutrient homeostasis, ultimately leading to decreased photosynthesis, growth, and yield [3–5]. Given the increasing pressure on global food security, developing effective strategies to mitigate salinity stress is essential.

Onion (*Allium cepa* L.), a widely consumed vegetable of nutritional and medicinal importance, is moderately sensitive to salinity because of its shallow root system and limited ability to exclude sodium ions. Under salinity stress, onions experience reductions in plant height, bulb size, nutrient content, and overall productivity [6, 7]. Global onion production, which reached 83 million tons in 2012, faces challenges in semi-arid regions, where salinity affects 25–30% of agricultural land.

Enhancing plant tolerance to salinity involves multiple mechanisms, including osmotic regulation, antioxidative defenses, and nutrient uptake. Osmoprotectants such as sugars, proline, and polyols play a central role in alleviating stress by stabilizing cellular structures, reducing oxidative damage, and maintaining osmotic balance [8–10]. Sugars, in particular, serve as signaling molecules that regulate carbon metabolism, abscisic acid levels, and reactive oxygen species (ROS) scavenging, contributing to improved stress resilience [11–13].

Despite the well-documented role of sugars in mitigating abiotic stress, few studies have explored their role in onion under salinity stress. Previous research has demonstrated that exogenous sugar applications, such as glucose and sucrose, enhance plant growth, photosynthesis, and antioxidant capacity under various stress conditions [14, 15]. However, the comparative efficacy of reducing sugars like glucose (Glu), lactose (Lac), melibiose (Mel), and xylose (Xyl) remains poorly understood in onion plants.

We hypothesized that foliar application of reducing sugars—glucose, lactose, melibiose, and xylose—would differentially enhance salinity tolerance in onion plants by improving osmoprotection, reducing oxidative stress, and maintaining ion homeostasis under moderate and severe NaCl stress.

To test this hypothesis, we evaluated the effects of foliar application of reducing sugars on the physiological, biochemical, and growth responses of onion plants subjected to salinity stress. Specifically, the study quantified photosynthetic pigments, antioxidative enzyme activities, osmoprotectants, elemental composition, and volatile sulfur compounds. Additionally, the Estimated Daily Intake (EDI) of essential elements was assessed to explore potential human health implications.

By addressing these objectives, this study contributes to a better understanding of how specific reducing sugars influence salinity tolerance mechanisms in onions, providing insight for potential applications in salt-affected cultivation systems.

## Materials and methods

### Experimental design

A pot experiment was conducted at the Department of Agricultural Botany, Faculty of Agriculture, Ain Shams University, Egypt, to evaluate the effects of reducing sugars on salinity tolerance in onion (*Allium cepa* L., cv. Giza Red)—a cultivar moderately sensitive to salt stress. Seedlings were obtained from the Crops Research Institute, Agricultural Research Center, and transplanted into 45 plastic pots (40 cm in diameter) filled with a 2:1 clay-to-sand mixture. Each pot contained five plants, giving a total of 225 plants. The experiment followed a completely randomized design (CRD) with three replicates per treatment.

Thirty days after planting, the treatments were initiated. Plants were irrigated weekly with 0 mM NaCl (control), 75 mM NaCl (moderate stress), or 125 mM NaCl (severe stress). Foliar sprays of reducing sugars—glucose, lactose, melibiose, and xylose—were applied at 10 mM. This concentration was selected based on preliminary screening, where 10 mM represented the lowest effective dose that elicited physiological responses without causing visible phytotoxic symptoms such as necrosis, chlorosis, or growth inhibition. Control plants received tap water only. Sugar solutions were applied weekly using a hand sprayer to ensure uniform leaf coverage.

Fertilization followed the Ministry of Agriculture’s recommendations, using calcium superphosphate (15.5% P₂O₅, 3 g pot⁻¹) before planting, and ammonium nitrate (33.5% N, 2.8 g pot⁻¹) plus potassium sulfate (48% K₂O, 0.5 g pot⁻¹) applied in two equal doses at the first and fourth irrigations. After 60 days of treatment, plants were harvested for physiological, biochemical, and elemental analyses.

### Instrumental analysis

#### Chemicals

All chemicals used in this study were of analytical grade (≥ 97% purity) and procured from reputable suppliers. Glucose, lactose, melibiose, xylose, quercetin, gallic acid, lysine, proline, catechol, guaiacol, bovine serum albumin, phenylalanine, 3,5-dinitrosalicylic acid, ninhydrin, thiobarbituric acid, trichloroacetic acid, Coomassie Brilliant Blue G-250, and the Folin–Ciocalteu reagent were purchased from Sigma-Aldrich S.p.A. (Milan, Italy). Other reagents, including sodium chloride, aluminum chloride, sodium carbonate, sodium hydroxide, sodium nitrite, potassium phosphate, potassium chloride, sodium acetate, ethanol (99.9%), methanol (99.9%), acetone (100%), hydrochloric acid (36%), hydrogen peroxide (50%), phosphoric acid, and toluene, were obtained from Piochem (Egypt).

For heavy-metal analysis, Suprapur^®^ concentrated nitric acid (HNO₃, 65% w/w) and Emsure^®^ hydrogen peroxide (H₂O₂, 30%) were purchased from Merck (Germany). Deionized water was produced in the laboratory using a purification system equipped with a Q-POD Element integrated with a Merck Millipore Q^®^ Integral 5 (A10^®^) unit. Nitric acid solutions (2% v/v) were prepared according to previously validated protocols [16, 17].

### Biochemical and physiological analyses

#### Photosynthetic pigments

Chlorophyll *a*, chlorophyll *b*, and carotenoid levels were determined using the acetone extraction method described by Costache et al. [18]. Fresh leaf samples (1 g) were homogenized in 100% acetone (50 mL), macerated for 24 h at 5 °C, and filtered through Whatman No. 1 filter paper. Absorbance was measured at 662, 645, and 470 nm using a spectrophotometer, and pigment concentrations were calculated using the following equations:

$$\mathrm{Chlorophyll}\;\mathrm a=11.75\;A_{662}-\;2.350\;A_{645}$$

$$\mathrm{Chlorophyll}\;\mathrm b\;=\;18.61\;A_{645}\;-\;3.960\;A_{662}$$

$$\:\mathrm{Carotenoids}=\frac{1000\:A_{470\:}-\:2.270\left(Chl\:a\right)-\:81.4\left(Chl\:b\right)}{227}$$

### Total phenolic and flavonoid contents

Total phenolic content (TP) was determined in a 70% ethanolic leaf extract using the Folin–Ciocalteu reagent, following Duca et al. [19], with gallic acid as the standard. Results were expressed as mg gallic acid equivalents (GAE) per 100 g FW.

In the same extract, total flavonoid content (TF) was measured using the aluminum chloride method, as described by Chang et al. [20], with quercetin as the standard, and results expressed as mg quercetin equivalents (QE) per 100 g FW.

### Total anthocyanin content

Total anthocyanin content was determined using the pH-differential method, as described by Giusti and Wrolstad [21]. Fresh onion samples were homogenized in acidified methanol (methanol : 1 N HCl, 95 : 5 v/v) and incubated at 4 °C for 24 h in the dark. The extract was filtered, and absorbance was measured at 510 nm and 700 nm in potassium chloride buffer (pH 1.0, 25 mM) and sodium acetate buffer (pH 4.5, 0.4 M).

Anthocyanin concentration was calculated as cyanidin-3-glucoside equivalents (mg CGE/100 g FW) using the formula:

$$\:\mathrm{Anthocyanin}\;\mathrm{content}=\frac{A\times MW\times DF\times1000}{\varepsilon\times Molar}$$

Where AA = [(A₅₁₀ − A₇₀₀) at pH 1.0] − [(A₅₁₀ − A₇₀₀) at pH 4.5], MW = 449.2 g mol⁻¹, DF = dilution factor, ε = 26,900 L mol⁻¹ cm⁻¹, and M = sample mass (g). Results were expressed as mg cyanidin-3-glucoside equivalents per 100 g fresh weight (mg CGE/100 g FW).

### Osmoprotectants

The levels of reducing sugars (RS), free amino acids (FAA), and proline were determined using standard protocols. RS was quantified in a leaf ethanolic extract using the 3,5-dinitrosalicylic acid (DNS) method, with glucose as the standard, and absorbance measured at 540 nm [22]. FAA was measured using the ninhydrin method, in which ethanolic extracts were reacted with ninhydrin reagent, heated at 100 °C for 15 min, and the absorbance read at 570 nm, with lysine as the standard [23].

Proline content was determined by homogenizing fresh leaves in 3% sulfosalicylic acid. The extract was reacted with ninhydrin reagent and glacial acetic acid at 100 °C for 1 h, cooled, and extracted with toluene. Absorbance was measured at 520 nm, and proline concentration was calculated using a standard calibration curve [24].

### Oxidative stress and antioxidant enzyme activities

#### Oxidative stress markers

Lipid peroxidation was assessed by quantifying malondialdehyde (MDA) levels using the thiobarbituric acid reactive substances (TBARS) assay as described by Gérard-Monnier et al. [25]. Fresh leaves were homogenized in 0.1% trichloroacetic acid (TCA), centrifuged at 12,000 × g for 15 min at 4 °C, and the supernatant was mixed with 0.5% thiobarbituric acid (TBA) in 20% TCA. The mixture was heated at 95 °C for 30 min, cooled, and absorbance was measured at 532 nm, with background correction at 600 nm. MDA content was calculated using an extinction coefficient of 155 mM⁻¹ cm⁻¹ and expressed as µM MDA g⁻¹ FW.

Hydrogen peroxide (H₂O₂) content was determined following Zhou et al. [26]. One millilitre of the same TCA extract was mixed with 10 mM potassium phosphate buffer (pH 7.0) and 1 M potassium iodide (KI). Absorbance was recorded at 390 nm, and results were expressed as mg H₂O₂ 100 g⁻¹ FW.

#### Antioxidant enzymes

Antioxidant enzyme activities were assessed using extracts prepared by homogenizing 500 mg of fresh leaves in 100 mM potassium phosphate buffer (pH 7.0) containing 1 mM EDTA and 1% PVPP, followed by centrifugation at 15,000 × g for 15 min at 4 °C. Total protein content, required for enzyme activity normalization, was determined using the Bradford assay [27], with bovine serum albumin (BSA) as the standard. Guaiacol peroxidase (POD, EC 1.11.1.7) activity was measured using 10 mM guaiacol and 5 mM H₂O₂, with absorbance recorded at 470 nm and expressed as µmol min⁻¹ mg⁻¹ protein, based on an extinction coefficient of 26.6 mM⁻¹ cm⁻¹ [28]. Polyphenol oxidase (PPO, EC 1.14.18.1) activity was determined using 10 mM catechol, with absorbance measured at 420 nm and expressed as µmol min⁻¹ mg⁻¹ protein, using an extinction coefficient of 3,400 mM⁻¹ cm⁻¹ [28]. Catalase (CAT, EC 1.11.1.6) activity was assessed by monitoring the decomposition of H₂O₂ at 240 nm, with results expressed as µmol H₂O₂ decomposed min⁻¹ mg⁻¹ protein, based on an extinction coefficient of 40 mM⁻¹ cm⁻¹ [29]. Phenylalanine ammonia-lyase (PAL, EC 4.3.1.5) activity was quantified by measuring the formation of trans-cinnamic acid at 290 nm, with one unit defined as an increase of 0.01 in absorbance per hour [30].

#### GC-MS analysis of volatile compounds

Volatile compounds were analyzed using gas chromatography–mass spectrometry (GC–MS) on an Agilent 7890B GC system coupled with a 5977 A mass selective detector, following the method of Adams [31]. Fresh onion samples were extracted with chloroform, filtered, and concentrated before injection. A DB-5MS capillary column (30 m × 0.25 mm × 0.25 μm) was used, with helium as the carrier gas at a constant flow rate of 3.0 mL min⁻¹. The GC temperature program was: 40 °C (1 min), ramped at 10 °C min⁻¹ to 200 °C (1 min), then 20 °C min⁻¹ to 220 °C (1 min), and finally 30 °C min⁻¹ to 320 °C (3 min). The injector and detector temperatures were set at 250 °C and 320 °C, respectively. The mass spectrometer operated in electron ionization (EI) mode at 70 eV, scanning a mass range of m/z 50–550 [32].

Compounds were identified by matching their mass spectra against the Wiley and NIST spectral libraries [33]. Identifications were confirmed based on both spectral similarity and retention index (RI) matching, with relative abundance expressed as total ion current (TIC) peak area percentages.

#### Elemental analysis

The mineral composition of onion samples was determined using inductively coupled plasma mass spectrometry (ICP–MS) following acid digestion, as described by Ghuniem [16]. Dried samples were finely ground, and 0.5 g of each was digested in 8 mL Suprapur^®^ nitric acid (HNO₃, 65% w/w) and 2 mL hydrogen peroxide (H₂O₂, 30%) using a Milestone Ethos UP microwave digestion system. The digestion program ramped to 200 °C over 15 min, was held for 15 min, and then cooled to below 80 °C before venting [14]. Digested samples were diluted to 50 mL with ultrapure deionized water (Milli-Q, 18.2 MΩ·cm) and filtered before analysis.

Elemental quantification was performed using a PerkinElmer NexION 2000 ICP–MS, equipped with an auto-sampler, Meinhard C concentric nebulizer, cyclonic spray chamber, and triple quadrupole technology. Calibration was performed using multi-element standard solutions (Merck, Germany) containing Cu, Zn, Fe, Mn, Ca, Na, K, P, and Mg, prepared in 2–3% HNO₃. A certified internal standard mixture (10 µg L⁻¹) of Bi, Ge, In, ⁶Li, Sc, Tb, and Y was used to correct for matrix effects [34–36]. Analytical precision was verified with a recovery rate of 95–105%, and detection limits were determined for each element. Results were expressed as mg kg⁻¹ dry weight (DW).

#### Estimation of daily intake

The potential dietary exposure to essential and trace elements through onion consumption was assessed by estimating the Provisional Tolerable Daily Intake (PTDI). The Estimated Provisional Tolerable Daily Intake (EPTDI) of each element was calculated using the equation:

$$\:EPTDI=\frac{F_c\times M_c}{B_W}\times10^{-3\:}(\text{m}\text{g}\:\text{k}\text{g}^{-1}\:\text{b}\text{w}\:\text{d}\text{a}\text{y}^{-1})$$

Where:

F_C_: Food consumption (g day⁻¹).

M_C_: Metal concentration (mg kg⁻¹).

B_W_: Average body weight (kg).

The elemental concentrations (MC) were derived from ICP-MS analysis, and food consumption data (FC) was based on the average daily onion consumption in Egypt, reported as 25 g day⁻¹ per person [37, 38]. An average body weight (BW) of 60 kg for adults was assumed, consistent with FAO/WHO recommendations [39].

The calculated EPTDI values were compared to the Tolerable Upper Intake Level (UL) and Acceptable Daily Intake (ADI) set by the Institute of Medicine and the Food and Nutrition Board (IOM-FNB, 2001) [40]. Elements exceeding their UL were flagged for potential health risks.

### Statistical analysis

The data were prepared for statistical analysis following Ramadan [41] and Ghadiriasli [42]. First, the dataset was normalized using z-score transformation to ensure comparability across different scales. A one-way analysis of variance (ANOVA) was performed to determine significant differences among treatments, followed by Duncan’s multiple range test to classify means and establish statistical significance at *p* < 0.05. Statistical analyses were conducted using XLSTAT (Addinsoft, France), and results are expressed as standard deviation (SD) of three independent replicates (*n* = 3).

Principal Component Analysis (PCA) was performed to identify patterns in the data and assess correlations between biochemical responses, plant growth parameters, and pigment levels under different salinity and sugar treatments. PCA was executed in XLSTAT, and components with eigenvalues > 1 were retained for interpretation.

All graphical representations, including bar plots with error bars and significance markers, were generated using Python’s Matplotlib and Seaborn libraries.

## Results

### Plant growth and physiological parameters

#### Growth metrics

**Fig. 1:**
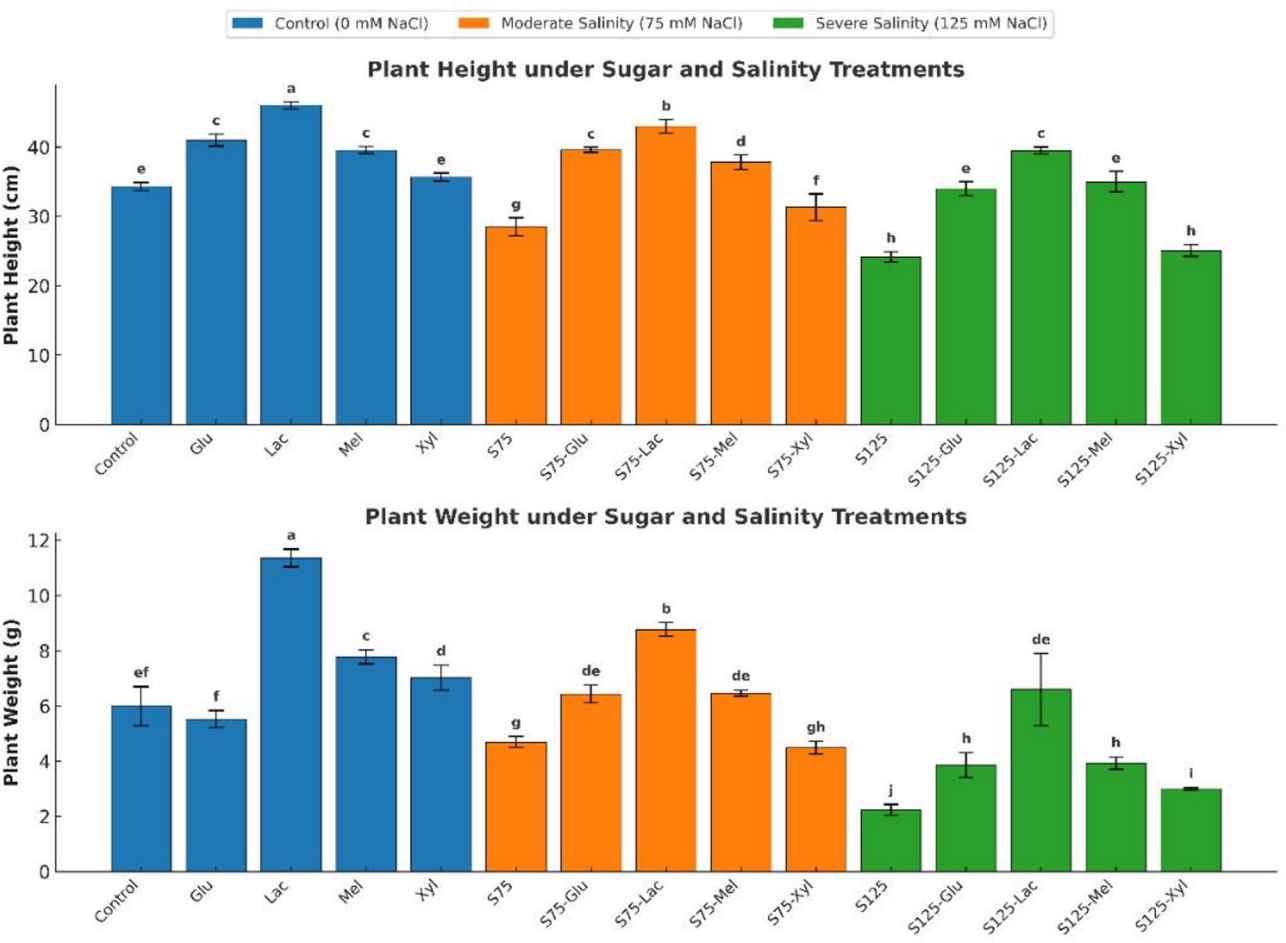
Effect of Salinity and Sugar Treatments on Plant Growth

Salinity stress significantly reduced onion plant height and fresh weight (*p* < 0.05), with greater reductions observed at 125 mM NaCl compared to 75 mM NaCl (Fig. 1). Specifically, plant height decreased from 34.33 cm in the control to 28.50 cm under 75 mM NaCl (16.91% reduction) and further to 24.17 cm under 125 mM NaCl (29.59% reduction). Similarly, fresh weight declined from 6.00 g (control) to 4.70 g and 2.24 g at 75 mM and 125 mM NaCl, representing respective decreases of 21.67% and 62.67%.

Plant height (cm) and fresh weight (g) of onion plants grown under different salinity levels (*0*,75, and 125 mM NaCl) with or without sugar treatments (*glucose*,*lactose*,*melibiose*, and *xylose*). Bars represent means ± standard deviation (*n* = 3). Different letters indicate significant differences (*p* < 0.05) based on Duncan’s test.

Foliar application of reducing sugars mitigated these negative effects of salinity. Lactose treatment significantly enhanced growth (*p* < 0.05), increasing plant height by 50.88% (to 43.00 cm) and fresh weight by 86.59% (to 8.77 g) compared with untreated plants under 75 mM NaCl. Under severe salinity (125 mM NaCl), lactose improved plant height by 63.54% (to 39.50 cm) and fresh weight by 194.64% (to 6.60 g) relative to the untreated control under the same salinity level. Melibiose and glucose treatments also improved growth parameters but to a lesser extent than lactose.

### Photosynthetic pigments

**Fig. 2:**
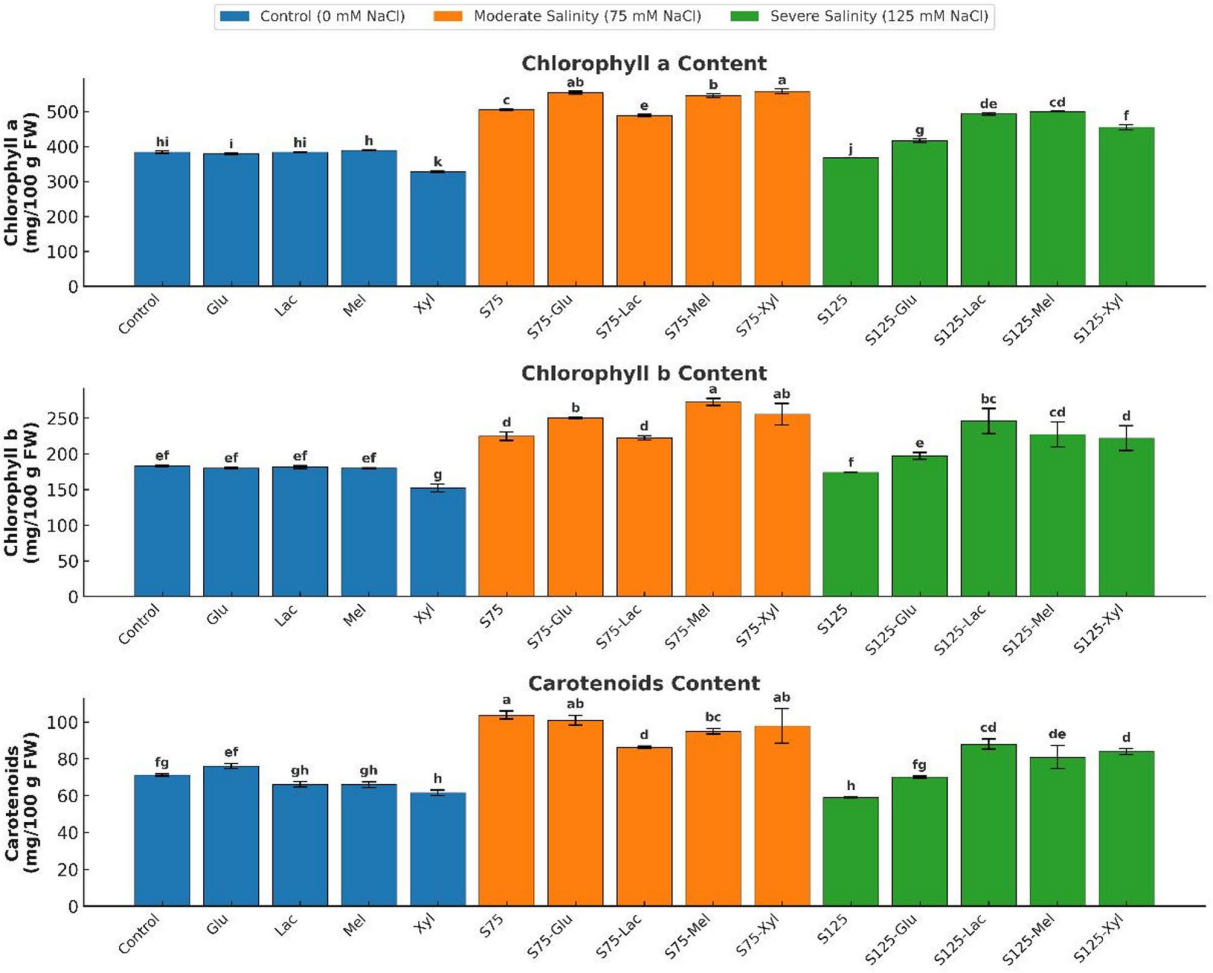
Effect of Salinity and Sugar Treatments on Photosynthetic Pigments

Exogenous sugars had minimal impact on photosynthetic pigment levels under normal conditions (Fig. 2). Glucose, lactose, and melibiose caused slight, non-significant variations in chlorophyll a (ranging from 378.67 to 389.25 mg 100 g FW⁻¹), chlorophyll b (179.72 to 181.22 mg 100 g FW⁻¹), and carotenoid concentrations (65.92 to 76.02 mg 100 g FW⁻¹) (*p* > 0.05). In contrast, xylose significantly reduced all pigment levels (*p* < 0.05), with chlorophyll a decreasing to 328.57 mg 100 g FW⁻¹, chlorophyll b to 151.86 mg 100 g FW⁻¹, and carotenoids to 61.51 mg 100 g FW⁻¹, indicating a potential inhibitory effect on pigment stability.

At moderate salinity (75 mM NaCl, S75), photosynthetic pigments increased relative to the non-saline control, with chlorophyll a, chlorophyll b, and carotenoids reaching 504.71 mg 100 g FW⁻¹, 224.59 mg 100 g FW⁻¹, and 103.74 mg 100 g FW⁻¹, respectively (*p* < 0.05). Exogenous sugar treatments further enhanced pigment accumulation under moderate salinity: xylose yielded the highest chlorophyll a content (558.04 mg 100 g FW⁻¹), while melibiose induced the greatest increase in chlorophyll b (272.65 mg 100 g FW⁻¹; *p* < 0.05). However, carotenoid levels did not significantly improve with sugar treatments (*p* > 0.05).

*Chlorophyll-a*,*chlorophyll-b*, and carotenoid (CA) content (mg/100 g FW) of onion plants under different salinity levels (*0*, 75, and 125 mM NaCl) with or without sugar treatments. Bars represent means ± standard deviation (*n* = 3). Different letters indicate significant differences (*p* < 0.05) based on Duncan’s test.

Under severe salinity (125 mM NaCl, S125), photosynthetic pigments declined significantly (*p* < 0.05), with chlorophyll a, chlorophyll b, and carotenoids dropping to 367.78 mg 100 g FW⁻¹, 174.08 mg 100 g FW⁻¹, and 58.94 mg 100 g FW⁻¹, respectively. Nevertheless, all sugar treatments significantly improved pigment retention (*p* < 0.05). Melibiose preserved chlorophyll a (499.95 mg 100 g FW⁻¹), lactose maintained chlorophyll b (245.93 mg 100 g FW⁻¹), and lactose also supported carotenoid retention (86.10 mg 100 g FW⁻¹). In contrast, glucose-treated plants exhibited lower pigment levels than other sugar treatments under severe salinity, with chlorophyll a at 416.57 mg 100 g FW⁻¹, chlorophyll b at 197.00 mg 100 g FW⁻¹, and carotenoids at 70.06 mg 100 g FW⁻¹.

### Total phenols (TP), total flavonoids (TF), and total anthocyanins (TA)

**Fig. 3:**
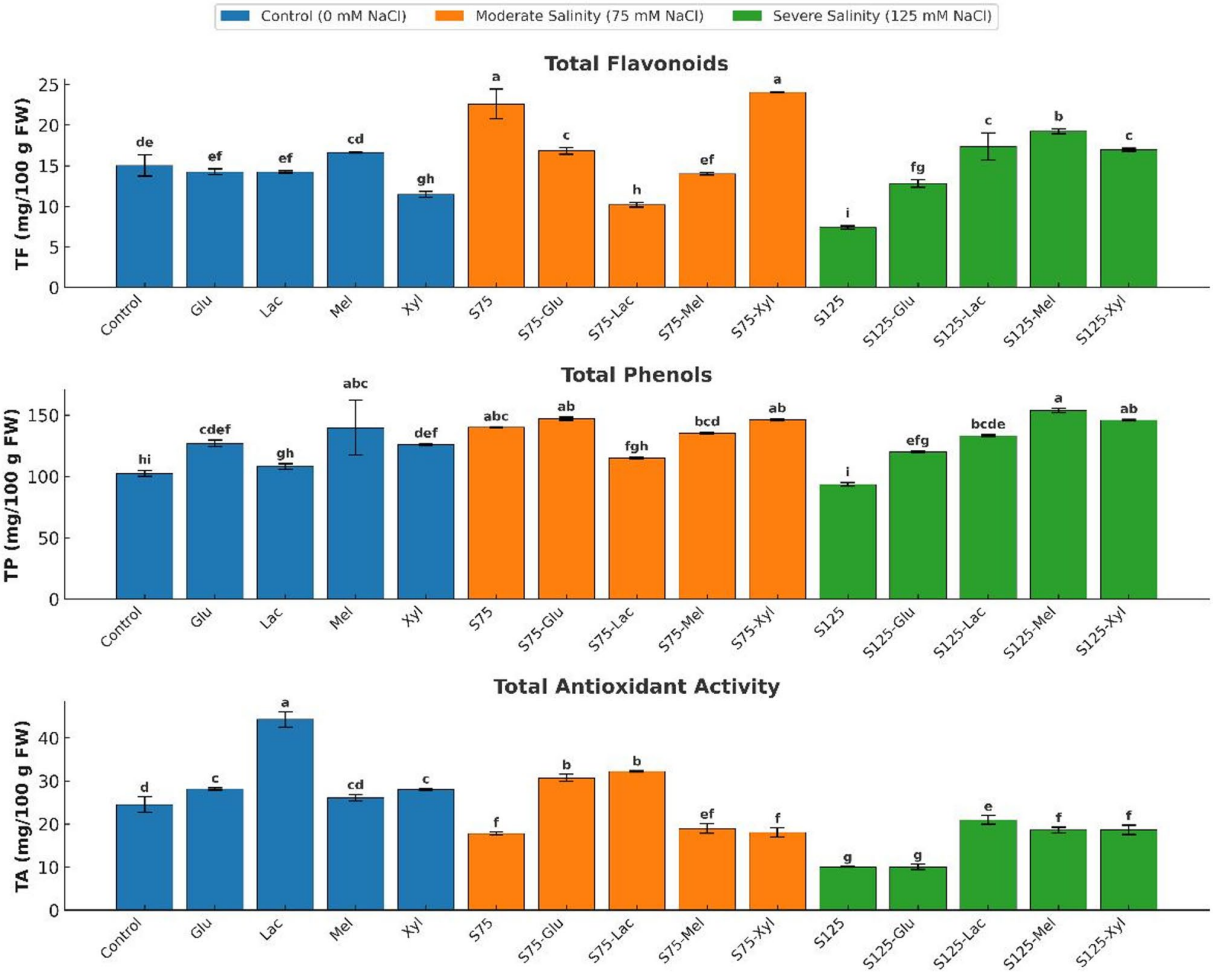
Effect of Salinity and Sugar Treatments on Secondary Metabolites

Exogenous sugars significantly influenced the accumulation of secondary metabolites in control plants (Fig. 3). Melibiose exhibited the strongest stimulatory effect, increasing total phenols to 139.78 mg GAE 100 g FW⁻¹ and total flavonoids to 16.64 mg QE 100 g FW⁻¹, compared with the control (102.29 mg GAE 100 g FW⁻¹ and 15.04 mg QE 100 g FW⁻¹, respectively; *p* < 0.05). Glucose and xylose also enhanced total phenolic content (*p* < 0.05), whereas lactose had no significant effect on phenols or flavonoids but significantly increased total anthocyanins to 44.29 mg CGE 100 g FW⁻¹, nearly double the control level (24.54 mg CGE 100 g FW⁻¹).

Salinity stress elicited a biphasic response in secondary metabolism. Moderate salinity (75 mM NaCl) significantly increased total phenols (140.07 mg GAE 100 g FW⁻¹) and flavonoids (22.61 mg QE 100 g FW⁻¹), reflecting activation of the phenolic defense pathway (*p* < 0.05). However, anthocyanin levels declined to 17.82 mg CGE 100 g FW⁻¹, suggesting partial pigment degradation under osmotic stress. Under moderate salinity, glucose and xylose maintained high phenolic levels, and xylose significantly elevated flavonoids to 24.04 mg QE 100 g FW⁻¹, while lactose notably enhanced anthocyanin content to 32.24 mg CGE 100 g FW⁻¹ (*p* < 0.05).

*Total Flavonoids (TF)*, total phenolic content (TP) and total anthocyanins (AT)content(mg/100 g FW)of onion plants under different salinity levels (*0*, 75, and 125 mM NaCl) with or without sugar treatments. Bars represent means ± standard deviation (*n* = 3). Different letters indicate significant differences (*p* < 0.05) based on Duncan’s test.

Under severe salinity (125 mM NaCl), all secondary metabolites were markedly suppressed (*p* < 0.05), with total phenols decreasing to 93.30 mg GAE 100 g FW⁻¹, flavonoids to 7.39 mg QE 100 g FW⁻¹, and anthocyanins to 10.05 mg CGE 100 g FW⁻¹. Among sugar treatments, melibiose proved most effective, elevating total phenols to 153.76 mg GAE 100 g FW⁻¹, while xylose maintained moderately high levels. Both melibiose and lactose significantly improved flavonoid and anthocyanin contents, with melibiose producing the highest anthocyanin accumulation (20.93 mg CGE 100 g FW⁻¹).

These findings highlight melibiose and lactose as key enhancers of secondary metabolism, potentially contributing to improved antioxidant capacity and photoprotection under saline stress.

### Osmoprotectant contents

**Fig. 4:**
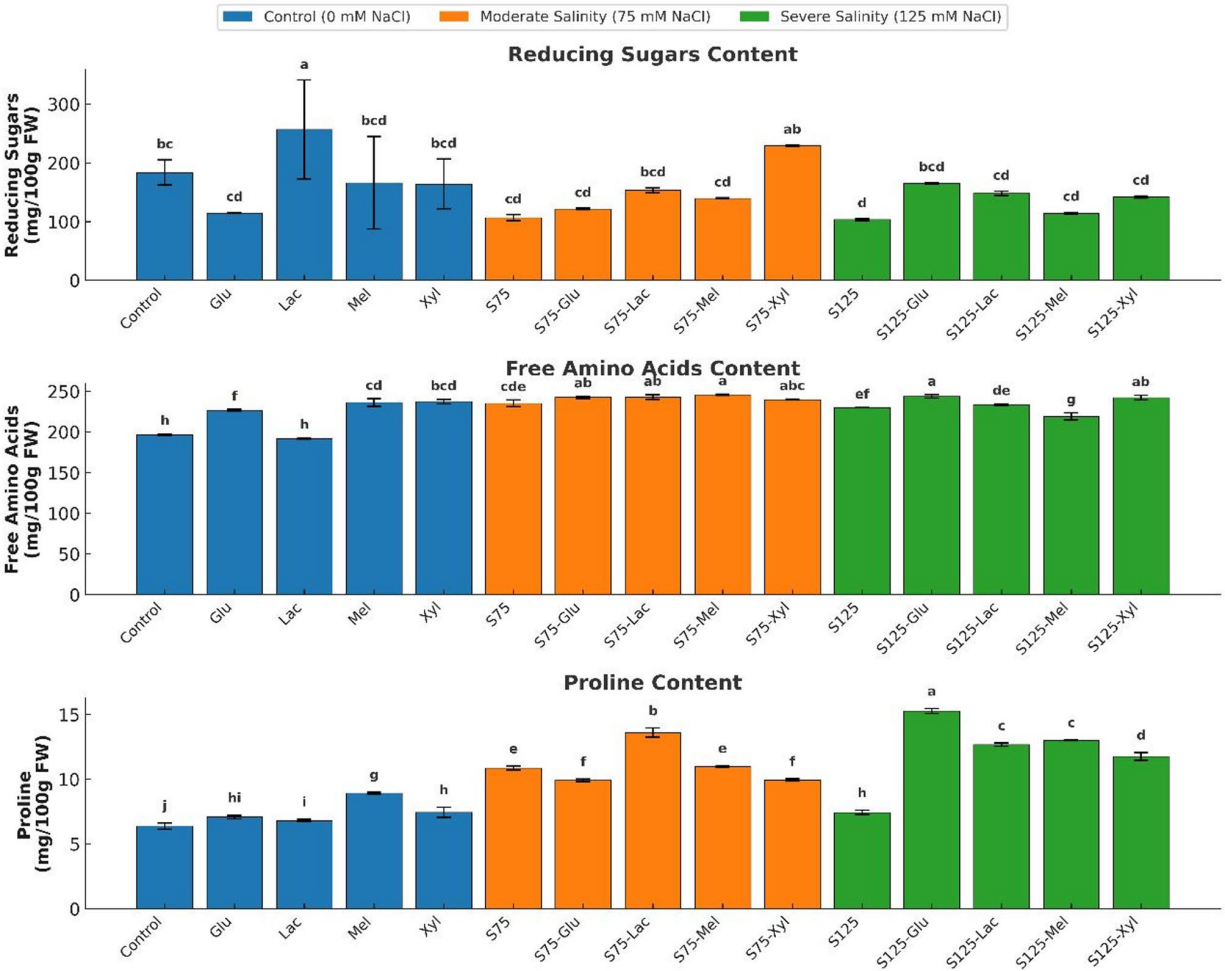
Effect of Salinity and Sugar Treatments on Osmoprotectant Accumulation

Exogenous sugars significantly influenced the accumulation of reducing sugars (RS), free amino acids (AA), and proline, showing distinct responses across salinity levels (Fig. 4). Under non-saline conditions, lactose markedly increased RS to 256.91 mg 100 g FW⁻¹ (*p* < 0.05), while glucose caused a significant decrease to 114.80 mg 100 g FW⁻¹ (*p* < 0.05). Melibiose and xylose showed no significant effect on RS (*p* > 0.05). All sugars significantly enhanced AA accumulation (*p* < 0.05), with xylose (237.45 mg 100 g FW⁻¹) and melibiose (236.25 mg 100 g FW⁻¹) recording the highest values. Proline content also increased under all sugar treatments (*p* < 0.05), with melibiose showing the greatest accumulation at 8.93 mg 100 g FW⁻¹.

At moderate salinity (75 mM NaCl), xylose significantly increased RS to 229.16 mg 100 g FW⁻¹ (*p* < 0.05), whereas glucose and melibiose induced moderate but non-significant increases (*p* > 0.05). AA accumulation rose significantly across all sugar treatments (*p* < 0.05), with melibiose exhibiting the highest level at 245.63 mg 100 g FW⁻¹. Lactose notably enhanced proline concentration to 13.60 mg 100 g FW⁻¹ (*p* < 0.05), while other sugars caused moderate, non-significant increases.

*Reducing sugars (RS)*, free amino acids *(AA)*, and proline (Pro) content (mg/100 g FW) in onion plants under different salinity levels (*0*, 75, and 125 mM NaCl) with or without sugar treatments. Bars represent means ± standard deviation (*n* = 3). Different letters indicate significant differences (*p* < 0.05) based on Duncan’s test.

Under severe salinity (125 mM NaCl), RS levels remained statistically unchanged across treatments (*p* > 0.05), indicating that high salinity restricted carbohydrate accumulation regardless of sugar supplementation. In contrast, glucose and xylose significantly increased AA content to 243.82 and 242.39 mg 100 g FW⁻¹, respectively (*p* < 0.05), while melibiose caused a reduction to 219.07 mg 100 g FW⁻¹ (*p* < 0.05), reflecting the metabolic cost of severe stress on nitrogen assimilation. Glucose markedly enhanced proline accumulation to 15.27 mg 100 g FW⁻¹ (*p* < 0.05), followed by melibiose (13.00 mg 100 g FW⁻¹) and lactose (12.67 mg 100 g FW⁻¹), whereas xylose-treated plants showed the lowest proline level (11.76 mg 100 g FW⁻¹; *p* < 0.05).

### Oxidative stress markers

**Fig. 5:**
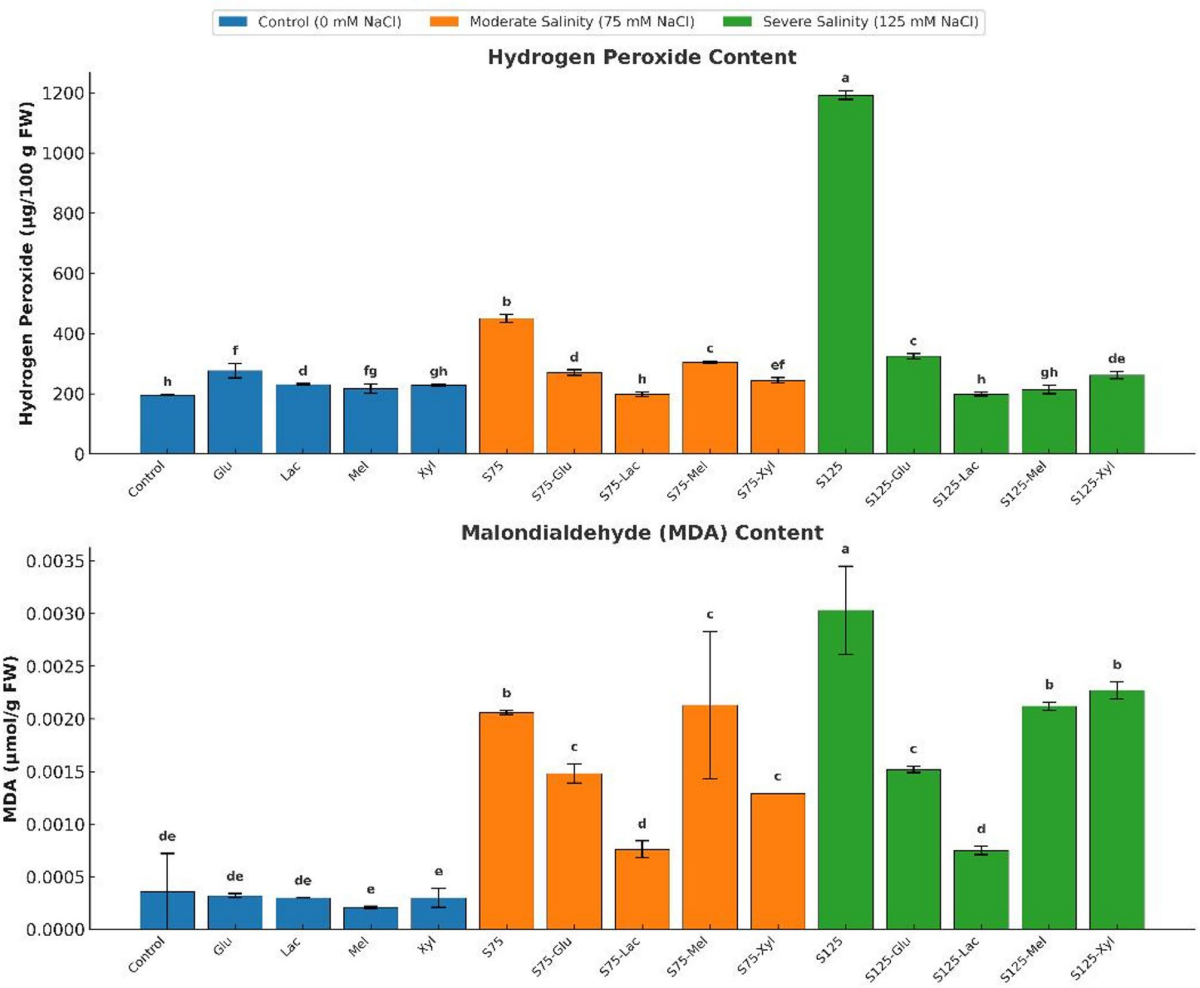
Effect of Salinity and Sugar Treatments on Oxidative Stress Markers

Exogenous sugar treatments significantly influenced oxidative stress indicators—hydrogen peroxide (H₂O₂) and malondialdehyde (MDA)—under both non-saline and saline conditions (Fig. 5).

Under non-saline conditions, glucose significantly increased H₂O₂ accumulation to 276.47 mg 100 g FW⁻¹ (*p* < 0.05), suggesting a mild oxidative response likely linked to enhanced metabolic activity. Lactose and xylose also induced significant increases, reaching 230.65 mg 100 g FW⁻¹ and 228.40 mg 100 g FW⁻¹, respectively, whereas melibiose caused a moderate but non-significant rise to 216.89 mg 100 g FW⁻¹. The control plants exhibited the lowest H₂O₂ concentration at 195.23 mg 100 g FW⁻¹. In contrast, MDA levels showed no significant variation among treatments (*p* > 0.05), remaining around 0.0003 µmol g FW⁻¹, indicating that lipid peroxidation was not induced by sugar application under normal conditions.

At moderate salinity (75 mM NaCl), all sugars significantly reduced H₂O₂ concentrations compared with the untreated saline control (450.11 mg 100 g FW⁻¹; *p* < 0.05). Lactose exhibited the most pronounced antioxidant effect, lowering H₂O₂ to 197.82 mg 100 g FW⁻¹, followed by xylose (244.28 mg 100 g FW⁻¹), glucose (270.02 mg 100 g FW⁻¹), and melibiose (304.65 mg 100 g FW⁻¹). For MDA, all sugars except melibiose significantly reduced lipid peroxidation relative to the control (0.0021 µmol g FW⁻¹). Lactose markedly decreased MDA to 0.00076 µmol g FW⁻¹, while xylose and glucose lowered it to approximately 0.0013 and 0.0015 µmol g FW⁻¹, respectively. Melibiose showed no significant difference from the untreated control (*p* > 0.05).

Hydrogen peroxide (H₂O₂) content (mg/100 g FW) and malondialdehyde (MDA) content (µmol/g FW) in onion plants under different salinity levels (*0*,* 75*, and 125 mM NaCl) with or without sugar treatments. Bars represent means ± standard deviation (*n* = 3). Different letters indicate significant differences (*p* < 0.05) based on Duncan’s test.

Under severe salinity (125 mM NaCl), exogenous sugars effectively mitigated oxidative stress, reducing H₂O₂ from 1192.30 mg 100 g FW⁻¹ in the untreated control to 198.19 mg 100 g FW⁻¹ (lactose), 213.82 mg 100 g FW⁻¹ (melibiose), 261.64 mg 100 g FW⁻¹ (xylose), and 324.57 mg 100 g FW⁻¹ (glucose; *p* < 0.05). Similarly, all sugars significantly lowered MDA compared with the control (0.0030 µmol g FW⁻¹). Lactose provided the strongest protection, reducing MDA to 0.00075 µmol g FW⁻¹, followed by melibiose and xylose (~ 0.002 µmol g FW⁻¹) and glucose (0.0015 µmol g FW⁻¹).

Overall, these findings demonstrate that exogenous sugars mitigate oxidative stress in onion plants under salinity, with lactose consistently showing the highest antioxidative capacity by reducing both H₂O₂ accumulation and lipid peroxidation.

### Antioxidant enzyme activities

Enzymatic activities of catalase (CAT), peroxidase (POD), polyphenol oxidase (PPO), and phenylalanine ammonia-lyase (PAL) were evaluated under all treatments.

Under non-saline conditions, sugar treatments generally enhanced enzyme activities. Lactose induced the highest PAL (9314 U mg⁻¹ protein) and PPO (26.34 U mg⁻¹ protein; *p* < 0.05), while melibiose maximized CAT (600.97 U mg⁻¹ protein; *p* < 0.05) and POD (5850.07 U mg⁻¹ protein; *p* < 0.05). Xylose and lactose moderately increased PAL and POD but were less effective for PPO, while xylose-treated plants exhibited the lowest CAT activity.

**Fig. 6:**
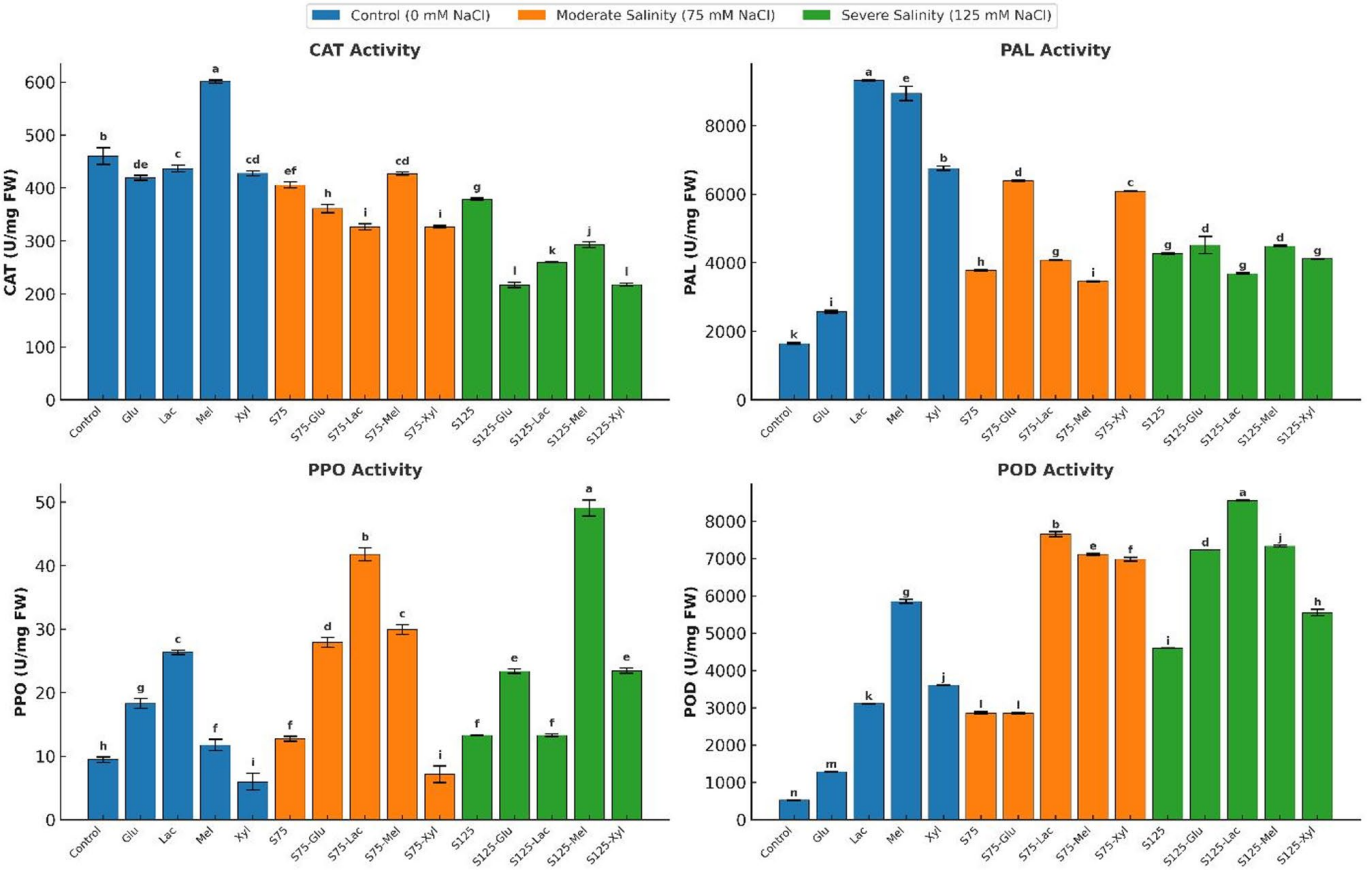
Effect of Salinity and Sugar Treatments on Antioxidant Enzyme Activities

At moderate salinity (75 mM NaCl, S75), untreated plants showed PAL activity of 3777.90 U mg⁻¹ protein. Glucose and xylose significantly enhanced PAL, reaching 6384.74 and 6087.81 U mg⁻¹ protein, respectively. Lactose significantly increased PPO to 41.77 U mg⁻¹ protein (*p* < 0.05), whereas xylose reduced PPO to 7.15 U mg⁻¹ protein (*p* < 0.05), suggesting possible inhibition. Lactose, melibiose, and xylose comparably increased POD activity, with lactose showing the highest stimulation. Melibiose maintained CAT activity, while other sugars caused slight reductions (Fig. 6).

*Phenylalanine ammonia-lyase (PAL)*,* catalase (CAT)*,* polyphenol oxidase (PPO)*, and peroxidase (POD) activities (U/mg protein) in onion plants under different salinity levels (*0*, 75, and 125 mM NaCl). Bars represent means ± standard deviation (*n* = 3). Different letters indicate significant differences (*p* < 0.05) based on Duncan’s test.

Under severe salinity (125 mM NaCl, S125), POD activity remained high, with lactose showing the strongest increase (8556.61 U mg⁻¹ protein; *p* < 0.05). PPO was markedly induced by melibiose (49.07 U mg⁻¹ protein), whereas lactose showed little effect. PAL activity increased under glucose and melibiose treatments to 4515.38 and 4486.21 U mg⁻¹ protein, respectively. CAT activity declined overall but was partially preserved by melibiose (292.91 U mg⁻¹ protein).

### Volatile sulfur compounds analysis

GC-MS identified 13 volatile sulfur-containing compounds in onion samples under control (0 mM NaCl, no sugars) and 75 mM NaCl treatments, with or without sugar supplementation (Table 1). The presence and relative abundance (Area %) of these compounds varied markedly across treatments, with some compounds uniquely detected under specific conditions.

**Table 1:** Effect of salinity and sugar treatments on volatile sulfur compounds

	Compounds	CAS	Area %					
			Control	S75	S75-Glu	S75-Lac	S75-Mel	S75-Xyl
1	(*E*)−1-Methyl-2-(prop-1-en-1-yl)disulfane	23838-19-9	N.D.	N.D.	3.85	2.81	2.91	N.D.
2	Sulfurous acid, hexyl nonyl ester	959311-73-0	N.D.	0.79	N.D.	N.D.	N.D.	N.D.
3	2,5-Dimethyl-thiophene	638-02-8	0.85	N.D.	N.D.	N.D.	N.D.	N.D.
4	4-Methylthiane, S-oxide		0.3	N.D.	N.D.	N.D.	N.D.	N.D.
5	*S*-Methyl methanethiosulphonate	2949-92-0	N.D.	N.D.	1.3	0.99	N.D.	N.D.
6	*cis*−2-Ethyl-3-methylthiophane	61568-37-4	N.D.	N.D.	1	0.52	N.D.	N.D.
7	(*E*)−1-(Prop-1-en-1-yl)−2-propyldisulfane	23838-21-3	N.D.	N.D.	3.04	1.72	N.D.	N.D.
8	1,2-Di((*E*)-prop-1-en-1-yl)disulfane	23838-23-5	N.D.	N.D.	1.93	N.D.	0.66	N.D.
9	2-Mercapto-3,4-dimethyl-2,3-dihydrothiophene	137363-86-1	N.D.	N.D.	3.02	N.D.	3.25	N.D.
10	3,4-Dimethylthiophene-2-thiol	153001-04-8	N.D.	N.D.	N.D.	N.D.	0.51	N.D.
11	3,4-dimethyl-2-(methyldisulfanyl)thiophene	126876-26-4	N.D.	N.D.	0.26	N.D.	N.D.	N.D.
12	Disulfide, di-tert-dodecyl	27458-90-8	0.62	N.D.	0.99	N.D.	0.72	N.D.
13	*tert*-Hexadecanethiol	25360-09-2	0.58	N.D.	N.D.	0.56	N.D.	N.D.

*Relative abundance (%) of volatile sulfur compounds in onion plants under control (0 NaCl + 0 sugars) and 75 mM NaCl with or without sugar treatments. The compounds were identified using GC-MS analysis*,* and their abundance is expressed as a percentage of the total detected volatiles. Bars represent mean values*,* with non-detected compounds assigned a value of zero for visualization purposes.*

Control plants exhibited low sulfur compound diversity (4 compounds), with two compounds uniquely detected: 2,5-dimethyl-thiophene (0.85%) and 4-methylthiane *S*-oxide (0.3%). Additionally, disulfidedi-*tert*-dodecyl (0.62%), and *tert*-hexadecanethiol (0.58%).

Under 75 mM NaCl stress, only sulfurous acid, hexyl nonyl ester (0.79%) was detected, indicating a substantial alteration in sulfur compound biosynthesis due to salinity stress.

Sugar supplementation altered the volatile sulfur profile notably. Glucose treatment (S75-Glu)) showed the highest sulfur compound diversity, with eight compounds detected at relatively high abundance: (*E*)−1-methyl-2-(prop-1-en-1-yl)disulfane (3.85%), (*E*)−1-(prop-1-en-1-yl)−2-propyldisulfane (3.04%),2-mercapto-3,4-dimethyl-2,3-dihydrothiophene (3.02%), 1,2-di((*E*)-prop-1-en-1-yl)disulfane (1.93%),*S*-methyl methanethiosulphonate (1.3%), cis-2-ethyl-3-methylthiophane(1%),disulfide, di-*tert*-dodecyl (0.99%) and 3,4-dimethyl-2-(methyldisulfanyl)thiophene (0.26%), all exclusively associated with this treatment.

Lactose treatment (S75-Lac) showed a lower sulfur compound accumulation (5 compounds), including (*E*)−1-methyl-2-(prop-1-en-1-yl)disulfane (2.81%), (*E*)−1-(prop-1-en-1-yl)−2-propyldisulfane (1.72%) *S*-methyl methanethiosulphonate (0.99%), *tert*-Hexadecanethiol (0.56%) and *cis*−2-ethyl-3-methylthiophane (0.52%).

Melibiose treatment (S75-Mel) exhibited a distinct profile (5 compounds), featuring 2-mercapto-3,4-dimethyl-2,3-dihydrothiophene (3.25%), (*E*)−1-methyl-2-(prop-1-en-1-yl)disulfane (2.91%), disulfide, di-*tert*-dodecyl (0.72%), 1,2-di((*E*)-prop-1-en-1-yl)disulfane (0.66%), and 3,4-dimethylthiophene-2-thiol (0.51%) present.

Xylose treatment (S75-Xyl) showed minimal detection of sulfur compounds, indicating a weaker impact on sulfur metabolism under salinity stress. Overall, the results suggest that glucose had the most pronounced effect on sulfur-based volatile biosynthesis, while xylose exhibited the weakest influence.

### Elemental composition

#### Macro-elements

**Fig. 7:**
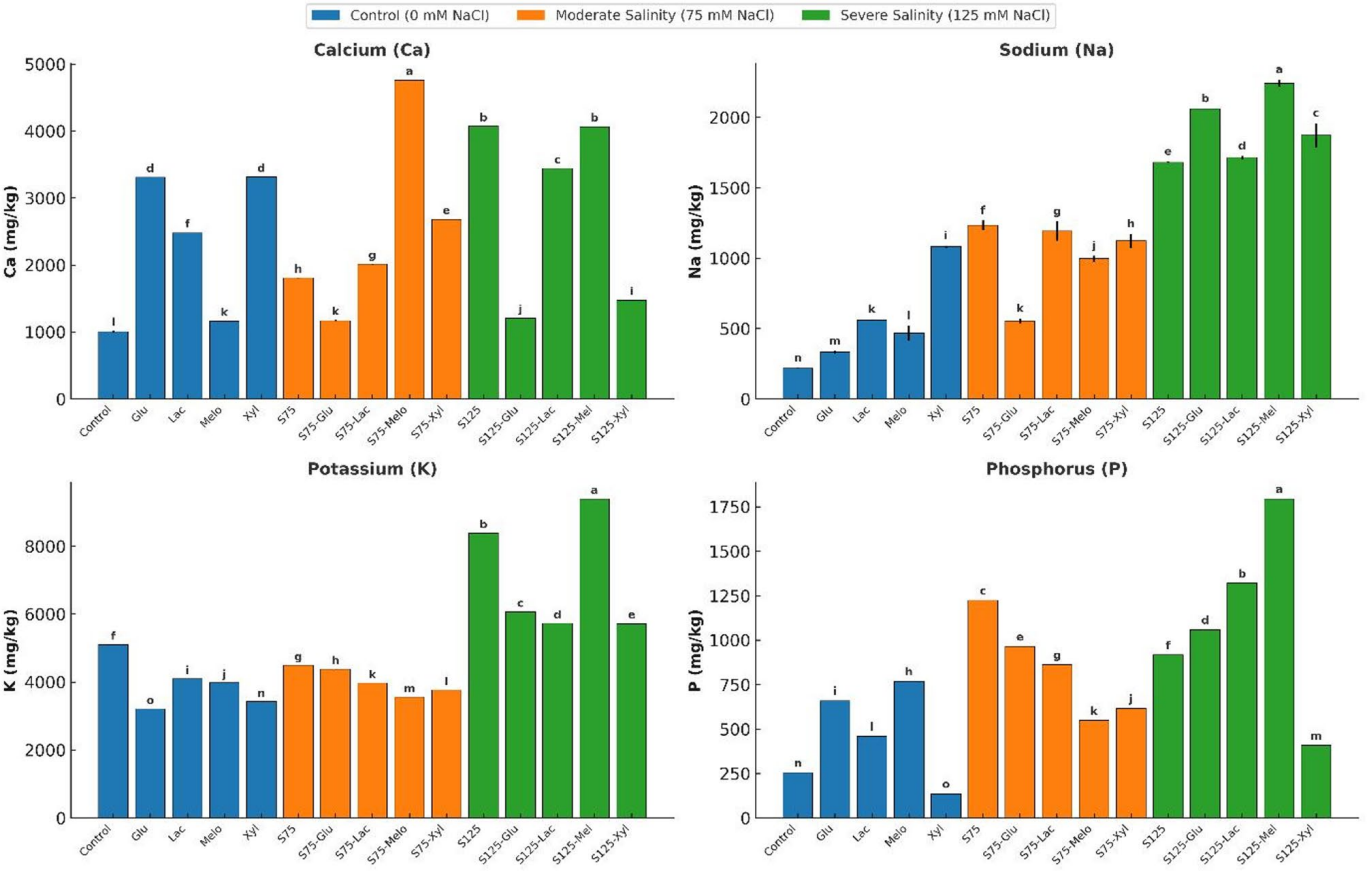
Effect of Salinity and Sugar Treatments on Elemental Composition

The impact of exogenous sugars and salinity on macro-element concentrations in onion plants was evaluated by measuring Ca, Na, K, and P under various treatments (Fig. 7). Control plants showed baseline levels of 1004.85 mg kg⁻¹ Ca, 217.83 mg kg⁻¹ Na, 5101.30 mg kg⁻¹ K, and 254.32 mg kg⁻¹ P.

Under non-saline conditions, exogenous sugars influenced element uptake significantly. Calcium levels increased with glucose (3310.92 mg kg⁻¹, *p* < 0.05), lactose (2478.33 mg kg⁻¹, *p* < 0.05), and xylose (3315.33 mg kg⁻¹, *p* < 0.05), while melibiose had a smaller effect (1163.34 mg kg⁻¹). Sodium rose significantly with xylose (1080.95 mg kg⁻¹) and lactose (561.36 mg kg⁻¹), while glucose maintained the lowest Na level (331.97 mg kg⁻¹). Potassium decreased under all sugar treatments, with glucose showing the lowest concentration (3208.60 mg kg⁻¹). Phosphorus increased notably with melibiose (769.19 mg kg⁻¹) and glucose (659.93 mg kg⁻¹), but declined with xylose (136.31 mg kg⁻¹).

*Calcium (Ca)*,*Sodium (Na)*,*Potassium (K)*, and Phosphorus (P) concentrations (mg/kg) in onion plants subjected to different salinity levels (*0*, 75, and 125 mM NaCl) with or without sugar treatments. Bars represent the mean concentrations per treatment. Different letters indicate significant differences (*p* < 0.05) based on Duncan’s test.

Salinity stress modulated nutrient accumulation differently. At moderate salinity (75 mM NaCl), calcium increased to 4072.81 mg kg⁻¹ but decreased under severe salinity (125 mM NaCl) to 1807.87 mg kg⁻¹, remaining higher than the control. Sodium and phosphorus followed similar patterns: sodium increased to 1681.65 mg kg⁻¹ at 75 mM NaCl before declining to 1234.44 mg kg⁻¹ at 125 mM NaCl, and phosphorus peaked at 1224.01 mg kg⁻¹ at 75 mM NaCl before falling to 916.81 mg kg⁻¹ at 125 mM NaCl. Potassium decreased under moderate salinity (4493.00 mg kg⁻¹) but rose substantially under severe stress (8386.99 mg kg⁻¹).

Under saline conditions, sugar treatments elicited variable responses. Melibiose elevated calcium most strongly, reaching 4761.81 mg kg⁻¹ at 75 mM NaCl and 4065.47 mg kg⁻¹ at 125 mM NaCl (*p* < 0.05). Sodium accumulation was reduced by all sugars at moderate salinity, particularly by glucose (554.55 mg kg⁻¹) and melibiose (996.78 mg kg⁻¹). However, sodium increased under all sugar treatments at severe salinity, especially with melibiose (2241.43 mg kg⁻¹), glucose (2061.61 mg kg⁻¹), and xylose (1873.08 mg kg⁻¹). Potassium and phosphorus showed parallel trends, decreasing with sugar treatments at 75 mM NaCl but increasing again at 125 mM NaCl, notably with melibiose (9391.52 mg kg⁻¹ and 1794.92 mg kg⁻¹, respectively).

### Micro-elements

Exogenous sugars and salinity markedly influenced microelement accumulation in onion plants (Table 2). Under non-saline conditions, Xyl produced the strongest stimulatory effect, markedly increasing Mg (563.15 mg kg⁻¹), Fe (1403.60 mg kg⁻¹), and Mn (11.61 mg kg⁻¹), while slightly elevating Cu (1.45 mg kg⁻¹). Lac significantly enhanced Zn (26.10 mg kg⁻¹), surpassing all other treatments, and moderately increased Mg (388.70 mg kg⁻¹) and Mn (5.89 mg kg⁻¹). Glu promoted balanced increases across elements, particularly Zn (15.64 mg kg⁻¹) and Cu (1.75 mg kg⁻¹), whereas Mel recorded the lowest overall micronutrient accumulation.

Under moderate salinity (75 mM NaCl), the untreated control (S75) exhibited elevated Mg (554.82 mg kg⁻¹) and Fe (269.56 mg kg⁻¹), suggesting ionic adjustment in response to stress. Sugar supplementation further enhanced uptake, with Mel displaying the most pronounced effect, sharply increasing Fe (2597.95 mg kg⁻¹) and Mn (39.32 mg kg⁻¹). Lac significantly improved Fe (615.66 mg kg⁻¹) and Zn (18.95 mg kg⁻¹), while Xyl and Glu showed moderate responses.

**Table 2:** Effect of salinity and sugar treatments on microelement accumulation in onion plants

Treatment	Elements (mg kg^− 1^)					
	Mg	Fe	Zn	Mn	Cu	Ba
Control	271.03 ± 0.53^m^	120.59 ± 4.18^i^	8.54 ± 0.06^h^	3.40 ± 0.17^j^	1.15 ± 0.00^k^	5.00 ± 0.00^d^
Glu	337.33 ± 2.34^k^	131.11 ± 0.86^hi^	15.64 ± 0.15^e^	4.91 ± 0.01^g^	1.75 ± 0.01^f^	5.00 ± 0.00^d^
Lac	388.70 ± 1.44^h^	72.28 ± 1.49^jk^	26.10 ± 0.06^a^	5.89 ± 0.01^f^	1.32 ± 0.01^j^	5.00 ± 0.00^d^
Mel	249.27 ± 0.63^n^	60.39 ± 0.29^kl^	8.64 ± 0.04^h^	2.85 ± 0.01^k^	0.90 ± 0.00^m^	5.20 ± 0.00^d^
Xyl	563.15 ± 0.77^d^	1403.60 ± 0.60^c^	17.17 ± 1.07^d^	11.61 ± 0.03^c^	1.45 ± 0.00^i^	5.00 ± 0.00^d^
S75	554.82 ± 1.60^e^	269.56 ± 1.31^e^	12.72 ± 0.57^f^	7.73 ± 0.05^d^	1.32 ± 0.01^j^	5.25 ± 0.35^d^
S75-Glu	317.71 ± 1.56^l^	53.99 ± 0.15^l^	13.43 ± 0.06^f^	3.37 ± 0.04^j^	1.08 ± 0.01^l^	5.00 ± 0.00^d^
S75-Lac	539.20 ± 1.24^f^	615.66 ± 0.24^d^	18.95 ± 0.57^c^	6.04 ± 0.00^e^	1.69 ± 0.00^g^	5.00 ± 0.00^d^
S75-Mel	644.83^b^	2597.95 ± 0.6^a^	21.87 ± 0.18^b^	39.32 ± 0.01^b^	2.25 ± 0.00^d^	5.00 ± 0.00^d^
S75-Xyl	500.03 ± 1.82^g^	180.35 ± 0.07^f^	11.32 ± 0.07^g^	4.98 ± 0.01^g^	2.09 ± 0.00^e^	5.00 ± 0.00^d^
S125	384.71 ± 0.56^i^	50.87 ± 0.75^l^	9.19 ± 0.12^h^	2.95 ± 0.02^k^	1.67 ± 0.00^h^	5.00 ± 0.00^d^
S125-Glu	372.69 ± 2.94^j^	156.68 ± 2.59^g^	16.83 ± 0.55^d^	4.90 ± 0.03^g^	3.31 ± 0.00^a^	5.00 ± 0.00^d^
S125-Lac	605.80 ± 0.95^c^	83.15 ± 0.32^j^	10.96 ± 0.22^g^	4.49 ± 0.02^h^	2.50 ± 0.01^c^	9.70 ± 0.00^b^
S125-Mel	723.83 ± 0.67^a^	2008.76 ± 1.68^b^	15.38 ± 0.25^e^	46.56 ± 0.03^a^	2.52 ± 0.01^b^	11.50 ± 0.00^a^
S125-Xyl	382.96 ± 3.11^i^	135.65 ± 25.37^h^	15.41 ± 0.06^e^	3.85 ± 0.02^i^	1.09 ± 0.00^l^	8.70 ± 0.01^c^

Microelement concentrations (*Mg*,* Fe*,*Zn*,*Mn*,* Cu*, and Ba in mg/kg) in onion plants under different salinity levels (*0*, 75, and 125 mM NaCl) with or without sugar treatments. Data are presented as mean ± standard deviation (*n* = 3). Different letters indicate significant differences (*p* < 0.05) based on Duncan’s test.

At severe salinity (125 mM NaCl), differential trends were observed. Mel markedly boosted Fe (2008.76 mg kg⁻¹) and Mn (46.56 mg kg⁻¹), indicating strong ionic retention under stress. Lac enhanced Mg (605.80 mg kg⁻¹) and Cu (2.50 mg kg⁻¹) while inducing the highest Ba (9.70 mg kg⁻¹) after Mel (11.50 mg kg⁻¹). Glu achieved the greatest Cu accumulation (3.31 mg kg⁻¹), and Xyl maintained moderate levels across elements.

Overall, Mel exhibited the most potent effect on Fe and Mn accumulation under salinity, Lac promoted Zn and Ba enrichment, and Glu primarily influenced Cu homeostasis, while Xyl enhanced Mg uptake under non-saline and moderate salinity conditions. These patterns suggest sugar-specific modulation of micronutrient uptake and redistribution depending on stress intensity.

### Ion homeostasis ratios (Ca^2+^/Na^+^ and K^+^/Na^+^)

**Fig. 8:**
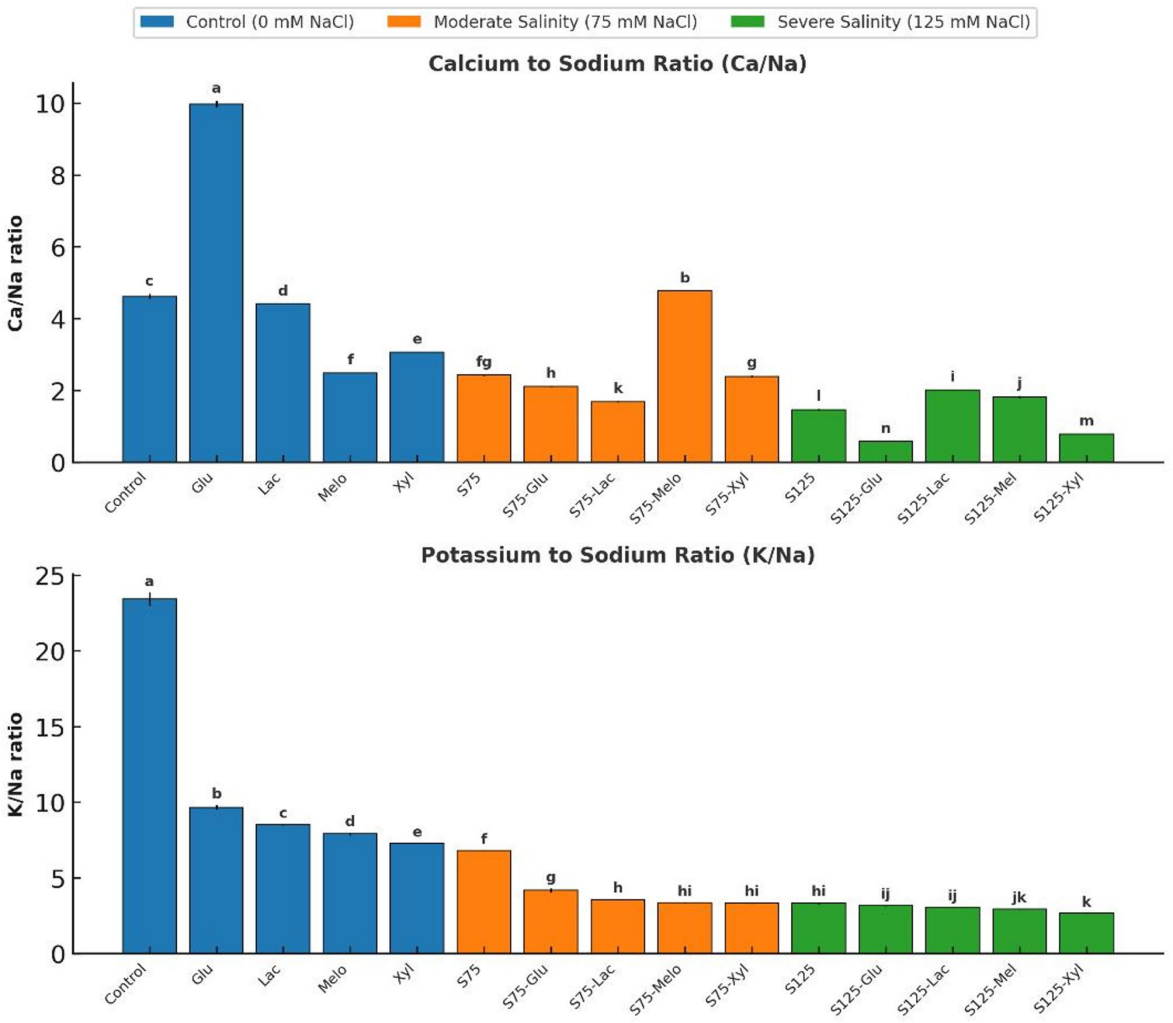
Effect of Salinity and Sugar Treatments on Ion Homeostasis (Ca^2+^/Na^+^ and K^+^/Na^+^)

Maintaining ion homeostasis, particularly the Ca²⁺/Na⁺ and K⁺/Na⁺ ratios, is vital for salinity tolerance as it reflects the plant’s capacity to limit Na⁺ buildup while conserving essential nutrients. Under control conditions, onion plants maintained a Ca²⁺/Na⁺ ratio of 4.61 and a K⁺/Na⁺ ratio of 23.42, indicating stable ion regulation (Fig. 8).

Under non-saline conditions, exogenous sugars altered these ratios in distinct ways. Glu markedly increased Ca²⁺/Na⁺ to 9.97, indicating improved Ca²⁺ retention and Na⁺ exclusion. In contrast, all sugars lowered the K⁺/Na⁺ ratio relative to control, with Xyl showing the lowest value (3.17), reflecting limited K⁺ retention under normal conditions.

Ca/Na and K/Na ratios in onion plants under different salinity levels (*0*, 75, and 125 mM NaCl) with or without sugar treatments. Lower ratios indicate increased sodium accumulation relative to calcium and potassium under salinity stress. Bars represent means ± standard deviation (*n* = 3). Different letters indicate significant differences (*p* < 0.05) based on Duncan’s test.

Salinity stress disrupted ionic equilibrium through excessive Na⁺ accumulation, sharply reducing both ratios. At 75 mM NaCl (S75), the Ca²⁺/Na⁺ ratio declined to 2.42, and K⁺/Na⁺ dropped to 2.67, indicating ion competition and nutrient imbalance. At 125 mM NaCl (S125), Ca²⁺/Na⁺ further decreased to 1.46, while K⁺/Na⁺ partially recovered to 6.79, suggesting partial K⁺ maintenance despite intensified Na⁺ stress.

Sugar supplementation alleviated these ionic imbalances to varying degrees. At S75, Mel maintained the highest Ca²⁺/Na⁺ ratio (4.77), followed by Xyl (2.39), while Glu (2.11) and Lac (1.69) were less effective. Glu showed the best performance in maintaining K⁺/Na⁺ (7.90), whereas Mel and Lac offered moderate improvement. At S125, Lac (2.00) and Mel (1.81) best preserved the Ca²⁺/Na⁺ ratio, while Glu (0.58) recorded the lowest value. Mel exhibited the highest K⁺/Na⁺ ratio (4.19), followed by Lac (3.34), indicating superior ionic regulation under severe salinity.

### Estimation of daily intake

To evaluate the nutritional implications and safety of onions grown under salinity and sugar treatments, the Estimated Provisional Tolerable Daily Intake (EPTDI) of essential elements was compared with the Recommended Dietary Allowance (RDA) and Tolerable Upper Intake Level (UL) where applicable [36, 37] (Tables 3 and 4). This evaluation ensures that mineral accumulation remains within safe dietary limits.

EPTDI was estimated for nine essential elements (Ca, Cu, Fe, Mg, Mn, P, K, Na, and Zn) across treatments. Most minerals remained within acceptable intake ranges; however, Fe and Mn exceeded the RDA in limited treatments. Specifically, Fe surpassed the RDA in four cases—onions treated with Xyl, Lac, and Mel—while Mn exceeded recommended levels only in Mel-treated samples.

For Ca (RDA: 1000 mg day⁻¹), estimated intake ranged from 4.99% in control plants to 16.46% in Xyl under non-saline conditions. Glu (16.44%) and Lac (12.30%) also showed elevated intake. Moderate salinity (75 mM NaCl) further increased Ca intake, reaching 20.22% in the salinity-only group and peaking at 23.64% with Mel. Under severe salinity (125 mM NaCl), intake slightly declined but remained above control levels, ranging from 5.99% in Glu to 20.18% in Mel, with Lac also showing high retention (17.09%).

For Cu (RDA: 0.9 mg day⁻¹), intake varied from 4.98% in Mel to 9.63% in Glu under non-saline conditions, with the control at 6.33%. Under moderate salinity, Cu remained within safe limits, peaking at 12.42% in Mel and 11.55% in Xyl. Severe salinity slightly elevated Cu intake, reaching 18.25% in Glu, followed by Mel (13.92%) and Lac (13.80%).

For Fe (RDA: 18 mg day⁻¹), intake ranged from 16.66% in Mel to 36.16% in Glu under control conditions. Xyl treatment resulted in exceptionally high Fe uptake (387.08%). Under moderate salinity, Fe intake increased further, exceeding safe levels in Mel (716.46%) and Lac (169.79%), while the salinity-only group reached 74.34%. Under severe salinity, Mel remained elevated (553.97%), while Glu showed moderate values (43.21%).

Fe intake in onions treated with Xyl, S75-Lac, S75-Mel, and S125-Mel exceeded the UL (45 mg day⁻¹), reaching 154.83%, 67.92%, 286.58%, and 221.59% of the UL, respectively, indicating potential overaccumulation.

**Table 3:** Estimated provisional tolerable daily intake (EPTDI) of essential elements in onion samples

Samples	Treatment								
	Ca	Na	K	*P*	Mg	Cu	Fe	Mn	Zn
**Control**	4.99%	0.72%	8.44%	1.80%	3.20%	6.33%	33.26%	8.45%	3.85%
**Glu**	16.44%	1.09%	5.31%	4.68%	3.99%	9.63%	36.16%	12.19%	7.06%
**Lac**	12.30%	1.86%	6.77%	3.26%	4.59%	7.29%	19.93%	14.62%	11.78%
**Mel**	5.78%	1.55%	6.58%	5.46%	2.95%	4.98%	16.66%	7.09%	3.90%
**Xyl**	16.46%	3.58%	5.67%	0.97%	6.66%	8.03%	387.08%	28.82%	7.75%
**S75**	20.22%	5.57%	7.43%	8.68%	6.56%	7.29%	74.34%	19.19%	5.74%
**S75-Glu**	5.81%	1.84%	7.25%	6.84%	3.76%	5.96%	14.89%	8.37%	6.06%
**S75-Lac**	9.99%	3.95%	6.57%	6.12%	6.37%	9.34%	169.79%	14.98%	8.55%
**S75-Mel**	23.64%	3.29%	5.89%	3.89%	7.62%	12.42%	716.46%	97.60%	9.87%
**S75-Xyl**	13.32%	3.72%	6.23%	4.38%	5.91%	11.55%	49.74%	12.36%	5.11%
**S125**	8.97%	4.09%	13.88%	6.50%	4.55%	9.24%	14.03%	7.33%	4.147%
**S125-Glu**	5.99%	6.82%	10.03%	7.51%	4.40%	18.25%	43.21%	12.16%	7.59%
**S125-Lac**	17.09%	5.68%	9.48%	9.37%	7.16%	13.80%	22.93%	11.15%	4.95%
**S125-Mel**	20.18%	7.42%	15.54%	12.73%	8.56%	13.92%	553.97%	115.58%	6.94%
**S125-Xyl**	7.31%	6.19%	9.47%	2.90%	4.53%	5.99%	37.41%	9.56%	6.95%

**Table 4:** Estimated provisional tolerable daily intake (EPTDI) of iron and manganese relative to the tolerable upper intake level (UL)

Elements	Treatments			
	Xyl	S75-Lac	S75-Mel	S125-Mel
Fe	154.834%	67.915%	286.583%	221.589%
Mn				21.015%

For Mg (RDA: 420 mg day⁻¹), estimated intake ranged from 2.95% in Mel to 6.66% in Xyl, with control at 3.20%. Under moderate salinity, values remained safe, peaking at 7.62% in Mel versus 6.56% in salinity-only treatment. Severe salinity showed similar trends, with intake ranging from 4.40% in Glu to 8.56% in Mel, and notable retention in Lac (7.16%).

For Mn (RDA: 2.3 mg day⁻¹), intake ranged from 7.09% in Mel to 28.82% in Xyl, with the control at 8.45%. At moderate salinity, Mel peaked at 97.60%, still within safe limits, but under severe salinity, Mel exceeded the RDA (115.58%) and reached 21.02% of the UL (11 mg day⁻¹).

For P (RDA: 700 mg day⁻¹), intake ranged from 0.97% in Xyl to 5.46% in Mel under control conditions. Moderate salinity increased P intake to 8.68% in the salinity-only treatment, while sugar supplementation slightly lowered values. Under severe salinity, Mel (12.73%) recorded the highest intake, followed by Lac (9.37%) and Glu (7.51%), whereas Xyl remained lowest (2.90%).

For K (RDA: 3400 mg day⁻¹), estimated intake varied from 5.31% in Glu to 8.44% in the control group. At moderate salinity, intake was relatively stable, peaking at 7.43% in the salinity-only group, with sugars slightly lowering levels. Under severe salinity, K intake increased to 15.54% in Mel and 13.88% in the salinity-only group, with other sugars showing moderate rises.

For Na (RDA: 1500 mg day⁻¹), intake ranged from 0.72% in control to 3.58% in Xyl under non-saline conditions, with sugars causing moderate increases. Moderate salinity raised Na intake, peaking at 5.57% in salinity-only plants. Lac (3.95%) and Xyl (3.72%) contributed higher levels, while Glu remained lowest (1.84%). Under severe salinity, Na intake peaked at 7.42% in Mel, followed by Glu (6.82%) and Lac (5.68%).

For Zn (RDA: 11 mg day⁻¹), intake ranged from 3.85% in control to 11.78% in Lac under non-saline conditions, with all sugars enhancing Zn levels. Under moderate salinity, Mel (9.87%) showed the highest intake, while Xyl (5.11%) had the lowest. Under severe salinity, Zn intake stayed within safe limits, with Glu (7.60%) showing the highest retention, followed by Lac (4.95%) and salinity-only (4.15%).

### Overall evaluation of samples

**Fig. 9:**
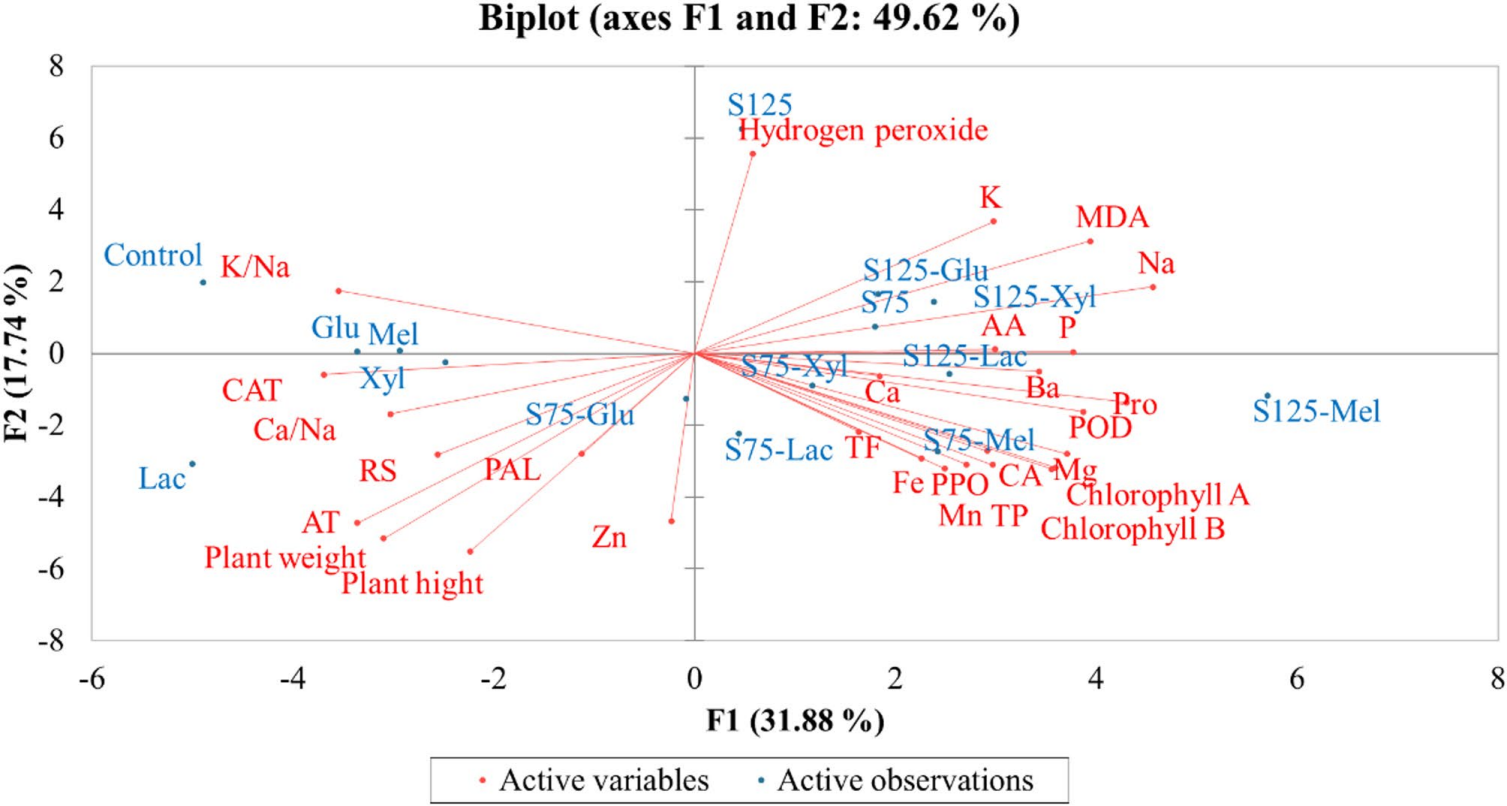
Principal Component Analysis (PCA) biplot of onion plant treatments

To elucidate the overall impact of treatments on onion plants, a Principal Component Analysis (PCA) was performed and visualized as a biplot (Fig. 9). The first two principal components (F1 and F2) accounted for 49.62% of the total variance across treatments. Component F1 (31.88%) primarily distinguished samples based on oxidative stress markers (MDA and H₂O₂) and biochemical variables such as proline, phenolics, and flavonoids. Component F2 (17.74%) separated treatments according to growth parameters (plant height and weight) and ion homeostasis ratios (K⁺/Na⁺ and Ca²⁺/Na⁺).

Control samples formed a distinct cluster characterized by high K/Na ratios and stable ionic balance, serving as a baseline. Under moderate salinity (S75), Lac and Mel treatments correlated strongly with improved growth (height and weight), higher catalase activity, and balanced Ca²⁺/Na⁺ ratios, indicating that stress mitigation under moderate salinity was mainly achieved through osmotic adjustment and enzymatic activation, which sustained ionic balance and minimized oxidative damage.

At severe salinity (S125), Lac and Mel were associated with elevated biochemical markers including proline, phenolics, and flavonoids, along with increased activities of antioxidative enzymes such as PPO and POD. The concurrent reduction in MDA and H₂O₂ levels suggests a transition in stress-defense mechanisms from osmotic regulation to structural reinforcement through phenolic biosynthesis and activation of the phenylpropanoid pathway, thereby enhancing antioxidative capacity and maintaining cellular integrity.

The PCA thus indicates a dual functional role for Lac and Mel: acting as osmoprotectants that improve ionic balance and catalase response under moderate salinity, and as biochemical modulators that strengthen structural defenses via phenolic metabolism under severe salinity.

Conversely, Glu and Xyl treatments under severe salinity clustered with higher oxidative stress and sodium accumulation, suggesting a limited capacity for ionic regulation and defense activation. Their rapid catabolism may restrict their osmoprotective efficacy under severe stress.

Overall, the PCA highlights Lac and Mel as the most effective sugar treatments for enhancing salinity tolerance in onion plants through coordinated regulation of ionic homeostasis, antioxidative defense, and structural metabolism.

## Discussion

Salinity stress profoundly affects plant growth and physiology by disrupting osmotic balance, ionic homeostasis, and inducing oxidative stress, which impair metabolic functions and reduce productivity [43–45]. In our study, onion plants exposed to salinity exhibited marked declines in growth, altered amino acid and sugar profiles, elevated oxidative stress markers, and disrupted nutrient uptake, consistent with prior reports of salinity’s multifaceted impact [46–48].

### Comparative roles of different sugars in stress mitigation

Our findings indicate that foliar application of specific sugars—glucose, lactose, melibiose, and xylose at 10 mM—can enhance onion tolerance to salinity stress by reducing oxidative damage and improving growth parameters such as plant height, fresh weight, and photosynthetic pigment content. These effects were accompanied by elevated phenolic, flavonoid, and anthocyanin levels, which are known to contribute to improved stress resilience [49–52]. Sugars appear to function not only as osmolytes but also as signaling molecules that regulate metabolic and gene expression pathways involved in stress adaptation [53].

The observed benefits align with previous studies showing that soluble sugars support osmoprotection, cellular hydration, and ionic homeostasis under salt stress, contributing to membrane stability and sustained water uptake [54–57]. For example, trehalose enhances photosynthetic efficiency and stomatal conductance under salinity, supporting our conclusions about sugar-mediated osmotic adjustment [55–57]. Additionally, sugars influence the pentose phosphate pathway to generate NADPH, essential for scavenging reactive oxygen species (ROS) and limiting oxidative stress [58, 59].

Among the sugars tested, melibiose showed the greatest effect on osmoprotection, consistent with its role in raffinose-family oligosaccharide synthesis and carbohydrate translocation, which support stress tolerance [60, 61]. Xylose also demonstrated antioxidant benefits, likely through membrane stabilization and ROS modulation [62]. The structural properties of sugars—particularly their hydroxyl groups—may explain their protective effects, with lactose and melibiose containing more equatorial hydroxyl groups that enhance water structuring and biomembrane integrity [63, 64]. Sugars rich in galactose have additionally been associated with strong antioxidant capacity [65].

While lactose has been less explored in plant stress physiology, microbial studies provide insights into its potential roles in osmotic regulation, energy metabolism, and stress-responsive signaling. For instance, *Streptococcus thermophilus* regulates lactose metabolism via carbon catabolite repression (CCR), optimizing sugar utilization and osmotic stability, suggesting a possible osmoprotective role in plants [66]. Similarly, in *Trichoderma reesei*, a specific lactose permease (Trire2:3405) is essential for uptake, linking lactose metabolism to energy conservation and secondary metabolism modulation [67].

### Ionic homeostasis and energy metabolism

Sugar application significantly influenced essential nutrient uptake in onion plants, notably affecting Ca²⁺, Na⁺, K⁺, P, Mg²⁺, Cu, Fe, Mn, and Zn. The observed enhancement of K⁺/Na⁺ and Ca²⁺/Na⁺ ratios following sugar treatments suggests an improved ionic balance, consistent with prior studies that report sugars modulate ion transport and stabilize membranes under abiotic stress conditions [59, 68, 69].

Among the sugars tested, glucose exhibited the strongest capacity to maintain Ca²⁺/Na⁺ and K⁺/Na⁺ homeostasis. This is likely due to its rapid metabolic integration into glycolysis, which facilitates ATP production necessary for energy-dependent ion transport processes. Supporting this, microbial metabolism studies indicate that glucose, unlike lactose, enters central carbohydrate metabolism directly without requiring additional enzymatic processing, enabling faster ATP generation and thus enhancing salt stress adaptation [70]. The superior performance of glucose in sustaining membrane integrity and promoting osmotic adjustments underscores its vital role as a primary energy source supporting ion regulation [71].

In contrast, lactose contributed primarily to osmotic stability but showed a comparatively weaker influence on ion homeostasis. This difference may stem from lactose’s slower metabolic entry and distinct regulatory effects on carbohydrate metabolism. Nonetheless, evidence from microbial systems suggests that lactose plays a role in metabolic conservation and sugar signaling, implying it may still support cellular adaptation strategies under stress—particularly by enhancing secondary metabolite biosynthesis and antioxidant defenses [72, 73].

### Impact on secondary metabolism and antioxidant defense

Phenylalanine ammonia-lyase (PAL), a pivotal enzyme in the phenylpropanoid pathway, showed increased activity following sugar treatments, resulting in elevated phenolic and flavonoid levels. These secondary metabolites are critical for reactive oxygen species detoxification and enhancing stress tolerance. Our findings align with previous reports demonstrating that salicylic acid (SA) treatment enhances PAL activity, promoting phenolic biosynthesis and improving stress resilience in plants such as *Catharanthus roseus* and *Solanum nigrum* [74–76].

Principal Component Analysis revealed strong correlations between zinc accumulation, phenolic content, and PAL activity, especially in plants treated with lactose and melibiose. Zinc is known to stimulate secondary metabolite production, supporting the idea that sugar-Zn interactions contribute to the adaptive response under salinity stress [77].

Moreover, sugar treatments induced the biosynthesis of novel organosulfur compounds, including (*E*)−1-Methyl-2-(prop-1-en-1-yl) disulfane, which was exclusively detected in glucose-, melibiose-, and lactose-treated plants. These sulfur-containing compounds possess well-documented antioxidant, antifungal, and cytoprotective properties, suggesting that foliar sugar application may modulate sulfur metabolism as part of the plant’s integrated defense mechanisms against salinity [69, 71].

## Conclusion

This study demonstrates that exogenous application of reducing sugars significantly enhances onion (*Allium cepa* L.) tolerance to salinity stress by improving growth, photosynthetic stability, osmoprotection, oxidative stress reduction, and ion homeostasis. Among the sugars tested, lactose and melibiose were most effective, leading to superior fresh weight recovery, proline accumulation, and antioxidant enzyme activities. These treatments also influenced sulfur metabolism and mineral uptake, notably maintaining calcium and potassium levels under salinity, thereby mitigating sodium toxicity.

Assessment of Estimated Daily Intake confirmed that most mineral levels remained within safe consumption limits, with only isolated cases of iron and manganese exceeding recommended thresholds in specific treatments. These findings support the practical viability of sugar-based strategies for salinity mitigation in onion cultivation.

Overall, lactose and melibiose emerge as cost-effective, sustainable options to enhance salinity tolerance in onions while preserving nutritional quality and food safety. Future studies should focus on field-scale trials and elucidation of the molecular mechanisms driving sugar-mediated stress responses, facilitating broader agricultural adoption in saline environments.

## Data Availability

The datasets generated and/or analyzed during the current study are available from the corresponding author upon request.

## References

[1] Qadir M, Quillérou E, Nangia V, Murtaza G, Singh M, Thomas RJ, et al. Economics of salt-induced land degradation and restoration. Nat Resour Forum. 2014;38:282–95.

[2] Zaman M, Shahid SA, Heng L. Guideline for salinity assessment, mitigation and adaptation using nuclear and related techniques. Cham: Springer Nature; 2018. 10.1007/978-3-319-96190-3

[3] Hailu B, Mehari H. Impacts of soil salinity/sodicity on soil-water relations and plant growth in dry land areas: a review. J Nat Sci Res. 2021;12:1–10.

[4] Rengasamy P. World salinization with emphasis on Australia. J Exp Bot. 2006;57:1017–23. 16510516 10.1093/jxb/erj108

[5] Khattab HI, Sadak MS, Dawood MG, Elkady FMA, Helal NM. Foliar application of Esculin and Digitoxin improve the yield quality of salt-stressed flax by improving the antioxidant defense system. BMC Plant Biol. 2024;24(1):963. 10.1186/s12870-024-05626-z. 39402439 10.1186/s12870-024-05626-z PMC11476730

[6] Hanci F, Cebeci E. Comparison of salinity and drought stress effects on some morphological and physiological parameters in onion (Allium Cepa L.) during early growth phase. Bulg J Agric Sci. 2015;21:1204–10.

[7] Patel JA, Vekaria LC, Sakarvadia HL, Parmar KB, Ponkia HP. Effect of saline irrigation water on growth and yields of onion (*Allium cepa* L.) varieties. Int J Curr Sci. 2020;8:966–9.

[8] Chen H, Jiang JG. Osmotic adjustment and plant adaptation to environmental changes related to drought and salinity. Environ Rev. 2010;18:309–19.

[9] Jogawat A. Osmolytes and their role in abiotic stress tolerance. In: Flower RJ, editor. Abiotic Stress Tolerance in Plants. Hoboken: Wiley-Blackwell; 2019. p.91–104.

[10] El-Lethy SR, Sadak MS, Hanafy RS. Assessing the usefulness of Moringa Oleifera leaf extract and zeatin in enhancing growth, phytohormones, antioxidant enzymes and osmoprotectants of wheat plant under salinity stress. Egypt J Bot. 2024;64(3):183–96. 10.21608/ejbo.2024.252447.2587.

[11] Smeekens S, Ma J, Hanson J, Rolland F. Sugar signals and molecular networks controlling plant growth. Curr Opin Plant Biol. 2010;13:273–8. 10.1016/j.pbi.2009.12.002 20056477

[12] Yang Y, Guo Y. Elucidating the molecular mechanisms mediating plant salt-stress responses. New Phytol. 2022;217:523–39. 10.1111/nph.14920 29205383

[13] Dawood MG, El-Awadi MS, Sadak MS. Chitosan and its nanoform regulates physiological processes and antioxidant mechanisms to improve drought stress tolerance of vicia Faba plant. J Soil Sci Plant Nutr. 2024. 10.1007/s42729-024-01934-3.

[14] Chang B, Yang L, Cong W, Zu Y, Tang Z. Improved resistance to high salinity induced by trehalose is associated with ionic regulation and osmotic adjustment in *Catharanthus roseus*. Plant Physiol Biochem. 2014;77:140–8. 24589477 10.1016/j.plaphy.2014.02.001

[15] Upadhyaya H, Sahoo L, Panda SK. Molecular Physiology of Osmotic Stress in Plants. In: Rout G, Das A, editors. Molecular Stress Physiology of Plants. India: Springer; 2013. pp. 179–92.

[16] Ghuniem MM, Khorshed MA, Souaya ER. Method validation for direct determination of some trace and toxic elements in soft drinks by inductively coupled plasma mass spectrometry. Int J Environ Anal Chem. 2019;99:515–40.

[17] Ghuniem MM, Souaya ER, Khorshed MA. Optimization and validation of an analytical method for the determination of some trace and toxic elements in canned fruit juices using quadrupole inductively coupled plasma mass spectrometer. J AOAC Int. 2019;102:262–70. 10.5740/jaoacint.18-0022 29776455

[18] Costache MA, Campeanu G, Neata G. Studies concerning the extraction of chlorophyll and total carotenoids from vegetables. Rom Biotechnol Lett. 2012;17:7702–8.

[19] Duca A, Sturza A, Moacă EA, Negrea M, Lalescu VD, Lungeanu D, et al. Identification of Resveratrol as a bioactive compound of propolis and characterization of phenolic profile and antioxidant activity of ethanolic extracts. Molecules. 2019;24:3368. 31527469 10.3390/molecules24183368 PMC6766919

[20] Chang CC, Yang MH, Wen HM, Chern JC. Estimation of total flavonoid content in propolis by two complementary colorimetric methods. J Food Drug Anal. 2002;10:178–82.

[21] Giusti MM, Wrolstad RE. Characterization and measurement of anthocyanins by UV-visible spectroscopy. Curr Protoc Food Anal Chem. 2001;1:F1.2.1-F1.2.13.

[22] Negrulescu A, et al. Adapting the reducing sugars method with dinitrosalicylic acid to microtiter plates and microwave heating. J Braz Chem Soc. 2012;23:2176–82.

[23] Jayaraman J. Laboratory manual of biochemistry. New Delhi: Wiley Eastern Ltd; 1981.

[24] Friedman M. Applications of the ninhydrin reaction for analysis of amino acids, peptides, and proteins. J Agric Food Chem. 2004;52:385–406. 14759124 10.1021/jf030490p

[25] Gérard-Monnier D, Erdelmeier I, Régnard K, Moze-Henry N, Yadan JC, Chaudiere J. Reactions of 1-methyl-2-phenylindole with malondialdehyde and 4-hydroxyalkenals. Chem Res Toxicol. 1998;11:1176–83. 9778314 10.1021/tx9701790

[26] Zhou B, Wang J, Guo Z, Tan H, Zhu X. A simple colorimetric method for determination of hydrogen peroxide in plant tissues. Plant Growth Regul. 2006;49:113–8.

[27] Bradford MM. A rapid and sensitive method for the quantitation of microgram quantities of protein utilizing the principle of protein-dye binding. Anal Biochem. 1976;72:248–54. 942051 10.1016/0003-2697(76)90527-3

[28] Simões AN, Moreira SI, Mosquim PR, Soares NF, Puschmann R. Effects of storage temperature on Kale leaves. Acta Agron. 2015;37:101–7.

[29] Aeby H, Catalases. Methods Enzymol. 1984;2:673–84.

[30] He C, Hsiang T, Wolyn DJ. Activation of defense responses to Fusarium infection in *Asparagus densiflorus*. Eur J Plant Pathol. 2001;107:473–83.

[31] Adams RP. Identification of essential oil components by gas chromatography/mass spectrometry. J Am Soc Mass Spectrom. 2007;18:803–6.

[32] Babushok VI, Linstrom PJ, Zenkevich IG. Retention indices for frequently reported compounds of plant essential oils. J Phys Chem Ref Data. 2011;40(4):043101.

[33] Stein SE. Mass spectral reference libraries: an ever-expanding resource for chemical identification. Anal Chem. 2012;84:7274–82. 22803687 10.1021/ac301205z

[34] Wolf WR. Inductively coupled plasma mass spectrometry for trace element analysis in foods. Anal Bioanal Chem. 2005;382:420–7.

[35] Thomas R. Practical Guide to ICP-MS: A Tutorial for Beginners. 2nd ed. Boca Raton: CRC Press; 2008.

[36] Jarvis KE, Gray AL, Houk RS. Handbook of Inductively Coupled Plasma Mass Spectrometry. New York: Chapman and Hall; 1992.

[37] World Bank. Jobs or Privileges? Unleashing the Employment Potential of the Middle East and North Africa. Washington, DC: World Bank; 2014. Report No.: 88879-MNA. Available from: https://documents.worldbank.org/en/publication/documents-reports/documentdetail/713481468278941596/jobs-or-privileges-unleashing-the-employment-potential-of-the-middle-east-and-north-africa

[38] Mostafa A, Ismail O, Mohamed A, EL-Ssanosy H. Economic study to production and consumption of onion and tomato crops in Egypt. New Valley Journal of Agricultural Science. 2022;2(3):148–57.

[39] Food and Agriculture Organization of the United Nations (FAO), World Health Organization (WHO). Human vitamin and mineral requirements: Report of a Joint FAO/WHO Expert Consultation. Bangkok, Thailand, 21-30 September 1998. Rome: Food & Nutrition Division, FAO; 2001.

[40] Institute of Medicine. Dietary Reference Intakes: The Essential Guide to Nutrient Requirements. Washington, DC: The National Academies Press; 2006.

[41] Ramadan KMA, El-Beltagi HS, El-Mageed TAA, Saudy HS, Al-Otaibi HH, Mahmoud MAA. The changes in various physio-biochemical parameters and yield traits of Faba bean due to humic acid plus 6-benzylaminopurine application under deficit irrigation. Agronomy. 2023;13(5):1227.

[42] Ghadiriasli R, Mahmoud MAA, Wagenstaller M, van de Kuilen JW, Buettner A. Chemo-sensory characterization of aroma-active compounds of native oak wood in relation to their geographical origins. Food Res Int. 2021;150:110776. 34865791 10.1016/j.foodres.2021.110776

[43] Boscaiu M, Bautista I, Donat P, Lidon A, Llinares J, Lull C, et al. Plant responses to abiotic stress. Curr Opin Biotechnol. 2011;22:S130.

[44] Farooq M, Hussain M, Nawaz A, Lee DJ, Alghamdi SS, Siddique KH, et al. Seed priming improves chilling tolerance in chickpea by modulating germination metabolism, trehalose accumulation, and carbon assimilation. Plant Physiol Biochem. 2017;111:274–83. 27987472 10.1016/j.plaphy.2016.12.012

[45] Rady MM, Sadak MS, El-Bassiouny HMS, Abd El-Monem AA. Alleviation the adverse effects of salinity stress in sunflower cultivars using nicotinamide and α-tocopherol. Aust J Basic Appl Sci. 2011;5(10):342–55.

[46] Munns R, Gilliham M. Salinity tolerance of crops–what is the cost? New Phytol. 2015;208(3):668–73. 10.1111/nph.13519. 26108441 10.1111/nph.13519

[47] Zörb C, Geilfus CM, Dietz KJ. Salinity and crop yield. Plant Biol (Stuttg). 2019;21(S1):31–8. 10.1111/plb.12884. 30059606 10.1111/plb.12884

[48] Sadak MS, Abd El-Monem AA, El-Bassiouny HMS, Badr NM. Physiological response of sunflower (Helianthus annuus L.) to exogenous arginine and Putrescine treatments under salinity stress. J Appl Sci Res. 2012;8(10):4943–57.

[49] Yang Y, Guo Y. Unraveling salt stress signaling in plants. J Integr Plant Biol. 2018;60(9):796–804. 10.1111/jipb.12655. 29905393 10.1111/jipb.12689

[50] Couée I, Sulmon C, Gouesbet G, El Amrani A. Involvement of soluble sugars in reactive oxygen species balance and responses to oxidative stress in plants. J Exp Bot. 2006;57(3):449–59. 10.1093/jxb/erj027. 16397003 10.1093/jxb/erj027

[51] Keunen E, Peshev D, Vangronsveld J, Van den Ende W, Cuypers A. Plant sugars are crucial regulators of oxidative stress during abiotic stress exposure. Front Plant Sci. 2013;4:307. 10.3389/fpls.2013.00307. 23305614 10.1111/pce.12061

[52] Verslues PE, Kim YS. Protein kinases and phosphatases for ABA and osmotic stress signaling in plants. Curr Opin Plant Biol. 2017;40:24–30. 10.1016/j.pbi.2017.06.006.

[53] Gasser C, Faurie JM, Rul F. Regulation of lactose, glucose, and sucrose metabolisms in S. thermophilus. Food Microbiol. 2024;121:104487. 10.1016/j.fm.2023.104487. 38637064 10.1016/j.fm.2024.104487

[54] Ivanova C, Bååth JA, Seiboth B, Kubicek CP. Systems analysis of lactose metabolism in *Trichoderma reesei* identifies a lactose permease that is essential for cellulase induction. PLoS One. 2013;8(5):e62631. 10.1371/journal.pone.0062631. 23690947 10.1371/journal.pone.0062631 PMC3648571

[55] Portnoy T, Margeot A, Linke R, Atanasova L, Fekete E, Sándor E, Kubicek CP, Seiboth B. The CRE1 carbon catabolite repressor of T. reesei: A master regulator of carbon assimilation. BMC Genomics. 2011;12:269. 10.1186/1471-2164-12-269. 21619626 10.1186/1471-2164-12-269 PMC3124439

[56] Sadak MS. Mitigation of drought stress on fenugreek plant by foliar application of trehalose. Int J ChemTech Res. 2016;9(2):147–55.

[57] Elewa TA, Sadak MS, Dawood MG. Improving drought tolerance of Quinoa plant by foliar treatment of Trehalose. Agric Eng Int. 2017;Special:245–45.

[58] Saloheimo M, Paloheimo M, Hakola S, Pere J, Swanson B, Nyyssönen E, et al. Swollenin, a *Trichoderma reesei* protein with sequence similarity to the plant expansins, exhibits disruption activity on cellulosic materials. Eur J Biochem. 2002;269(16):4202–11. 10.1046/j.1432-1033.2002.03095.x. 12199698 10.1046/j.1432-1033.2002.03095.x

[59] Dawood MG, El-Awadi MES, Abdel-Baky YR, Sadak MS. Physiological role of ascobin on quality and productivity of sunflower plants irrigated with sodium chloride solution. Agric Eng Int CIGR J. 2017;Special issue:16–26.

[60] ElSayed AI, Rafudeen MS, Golldack D. Physiological aspects of raffinose family oligosaccharides in plants: protection against abiotic stress. Plant Biol (Stuttg). 2014;16(1):1–8. 10.1111/plb.12053. 23937337 10.1111/plb.12053

[61] Nishizawa-Yokoi A, Yabuta Y, Shigeoka S. The contribution of carbohydrates including raffinose family oligosaccharides and sugar alcohols to protection of plant cells against oxidative damage under abiotic stress conditions. Plant Signal Behav. 2008;3(11):1016–8. 10.4161/psb.3.11.6249. 19704439 10.4161/psb.6738 PMC2633762

[62] El-Bassiouny HMS, Abdallah MMS, El-Enany MAM, Sadak MS. Nano-zinc oxide and arbuscular mycorrhiza effects on physiological and biochemical aspects of wheat cultivars under saline conditions. Pak J Biol Sci. 2020;23:478–90. 10.3923/pjbs.2020.478.490. 32363833 10.3923/pjbs.2020.478.490

[63] Selmar D, Kleinwächter M. Influencing the product quality by deliberately applying drought stress during cultivation of medicinal plants. Ind Crops Prod. 2013;42:558–66. 10.1016/j.indcrop.2012.06.020.

[64] Vereyken IJ, Chupin V, Demel RA, Smeekens SCM, De Kruijff B. Fructans insert between the headgroups of phospholipids. Biochimica et Biophysica Acta (BBA). 2001;1510(1–2):307–20. 11342168 10.1016/s0005-2736(00)00363-1

[65] Unno H, Maeda Y. Effect of exogenous application of sugars on the salt tolerance of perennial ryegrass protoplasts. Biologia. 2008;63:204–6.

[66] Andrew M, Jayaraman G. Structural features of microbial exopolysaccharides in relation to their antioxidant activity. Carbohydr Res. 2020;487:107881. 31805426 10.1016/j.carres.2019.107881

[67] Geilfus CM. The pH of the apoplast: dynamic factor with functional impact under stress. Mol Plant. 2017;10(11):1371–86. 10.1016/j.molp.2017.09.018. 28987886 10.1016/j.molp.2017.09.018

[68] Maathuis FJ. Physiological functions of mineral macronutrients. Curr Opin Plant Biol. 2009;12(3):250–8. 10.1016/j.pbi.2009.04.003. 19473870 10.1016/j.pbi.2009.04.003

[69] Michalak A. Phenolic compounds and their antioxidant activity in plants growing under heavy metal stress. Pol J Environ Stud. 2006;15(4):523–30.

[70] Hänsch R, Mendel RR. Physiological functions of mineral micronutrients. Curr Opin Plant Biol. 2009;12(3):259–66. 10.1016/j.pbi.2009.05.006. 19524482 10.1016/j.pbi.2009.05.006

[71] Khan NA, Syeed S, Masood A, Nazar R, Iqbal N. Application of Salicylic acid increases contents of nutrients and antioxidative metabolism in Mungbean and alleviates adverse effects of salinity stress. Sci Hortic (Amsterdam). 2010;126(3):220–7. 10.1016/j.scienta.2010.07.017.

[72] Lancaster JE, Shaw ML. G-Glutamyl peptides in the biosynthesis of S-alk(en)yl-L-cysteine sulfoxides (flavour precursors) in onions (Allium Cepa L.) and other allium species. J Sci Food Agric. 1994;64(1):93–103. 10.1002/jsfa.2740640116.

[73] Martínez-Ballesta MC, Martínez V, Carvajal M. Osmotic adjustment, water relations and gas exchange in broccoli plants under drought stress and recovery. Aust J Plant Physiol. 2004;31(9):999–1008. 10.1071/PP04062.

[74] Anwar S, Yun BW. Interplay between sugar and hormone signaling pathways modulate PAL activity in plants. Plant Signal Behav. 2020;15(4):1754015. 10.1080/15592324.2020.1754015.

[75] Ramakrishna A, Ravishankar GA. Influence of abiotic stress signals on secondary metabolites in plants. Plant Signal Behav. 2011;6(11):1720–31. 10.4161/psb.6.11.17613. 22041989 10.4161/psb.6.11.17613 PMC3329344

[76] Bakry AB, Sadak MS, Abd El-Monem AA. Physiological aspects of tyrosine and Salicylic acid on morphological, yield and biochemical constituents of peanut plants. Pak J Biol Sci. 2020;23:375–84. 10.3923/pjbs.2020.375.384.

[77] Tewari RK, Kumar P, Sharma PN. Oxidative stress and antioxidative responses in young leaves of mulberry plants grown under Zn-deficiency. J Plant Nutr Soil Sci. 2008;171(5):572–80. 10.1002/jpln.200700095.

